# Targeted identification of new phaterpenes and elucidation of the relevant biosynthetic pathway in *Streptomyces phaeochromogenes* OSK-123

**DOI:** 10.1016/j.synbio.2026.01.010

**Published:** 2026-01-25

**Authors:** Xing Fan, Xin Zhang, Minguo Tang, Shihao Wei, Ziyue Guo, Fucai Ren, Jiaying Hao, Lingqi Hua, Lin Zhou, Jie Xu, Wei Huang, Qianjin Kang, Linquan Bai

**Affiliations:** a State Key Laboratory of Microbial Metabolism, School of Life Sciences and Biotechnology, Shanghai Jiao Tong University, Shanghai, 200240, China; b College of Life Science and Technology, Tarim University, Alar, 843300, Xinjiang, China; c Tianjin Institute of Industrial Biotechnology, Chinese Academy of Sciences, West 7th Avenue No. 32, 300308, Tianjin, China; d School of Pharmacy, Anhui Medical University, Anhui, 230032, China

**Keywords:** Sesquiterpenes, Phaterpenes, Biosynthetic pathway, Genome mining, *Streptomyces phaeochromogenes* OSK-123

## Abstract

Sesquiterpenes have highly diverse chemical structures and biological activities, resulting in considerable interest in terms of their application and exploration of new analogs. In this study, we identified a new cryptic bacterial terpenoid biosynthetic gene cluster (i.e., *pha*) in the genome of the soil microorganism *Streptomyces phaeochromogenes* OSK-123 (strain OSK-123). According to online BiG-FAM and sequence similarity network analyses, *pha* was revealed to span an approximately 18 kb DNA region with 14 open reading frames, which likely include sequences encoding enzymes catalyzing the production of new sesquiterpenes. To facilitate the identification of target compounds, strong constitutive promoters were incorporated into *pha*. Additionally, we conducted a comparative analysis of different fermentation extracts for wild-type and promoter-substituted strains as well as RT-qPCR and LC-ESI-MS analyses to efficiently detect and identify target compounds. An examination of spectroscopic data identified four new 6/5-fused bicyclic sesquiterpenoid compounds, designated as phaterpene A–D (compounds **1**–**4**). The terpene synthase PhaA catalyzed the formation of a six-membered ring sesquiterpene skeleton via heterologous expression in *Escherichia coli*. The complete *pha* sequence was incorporated into plasmid pLQ1512 and heterologously expressed in *Streptomyces albus* J1074 to elucidate the phaterpene biosynthetic pathway. Candidate modification genes were disrupted and functionally validated in *S. albus* J1074. The protoporphyrinogen/coproporphyrinogen oxidase PhaB was identified to catalyze the formation of a 6,5-fused bicyclic sesquiterpenoid scaffold. On the basis of these findings, the new phaterpene biosynthetic pathway was established. This research not only presented a practical approach for discovery of the targeted compounds through integration of multiple pipelines, but also enriched the understanding of chemical diversity and biosynthetic machinery of the new sesquiterpenes.

## 1 Introduction

Sesquiterpenes are commonly found in plants [1], animals [2], and microorganisms [3]. Their considerable structural versatility results in various scaffolds and stereochemical diversity, which underlies their broad biological activities and ability to interact with diverse molecular targets. Sesquiterpenes can be classified on the basis of the number of rings (e.g., linear, monocyclic, bicyclic, and tricyclic types), but they can also be categorized according to the characteristics of their carbon skeletons (e.g., bisabolane [4], humulane [5], and germacrane [6] types). Considering their diverse biological roles, sesquiterpenoids are economically important biomedical compounds used in multiple industries (e.g., pharmaceutical, cosmetic, and agriculture). Typical examples include artemisinin (antimalarial compound) [7], (−)-*epi*-α-bisabolol (cosmetic component) [8], and aspterric acid (herbicide) [9].

On the basis of the number of isoprene units, terpenoids can be structurally classified as hemiterpenes (C5), monoterpenes (C10), sesquiterpenes (C15), diterpenes (C20), sesterterpenes (C25), triterpenes (C30), and tetraterpenes (C40, e.g., carotenoids) [10]. The vast diversity of terpenoids is due to their complex and flexible biosynthetic pathways. All terpenoids, regardless of their structural complexity, are derived from the universal C5 precursors isopentenyl diphosphate (IPP) and dimethylallyl diphosphate (DMAPP), which are synthesized via the mevalonate (MVA) or methylerythritol phosphate pathway [11]; although evolutionarily distinct, both pathways involve the same universal precursors. Through sequential “head-to-tail” condensations, IPP and DMAPP yield key linear intermediates, including geranyl diphosphate (GPP, C10), farnesyl diphosphate (FPP, C15), and geranylgeranyl diphosphate (GGPP, C20). These intermediates are then cyclized, rearranged, or further modified by terpene synthases (TPSs) through carbocation-mediated mechanisms to generate diverse terpenoid scaffolds [12]. TPSs are usually grouped into Class I and Class II. Notably, Class I TPSs have separate DDxxD and NSE/DTE motifs that are used to select the diphosphate group, thereby triggering cyclization. Class II TPSs generate carbocations by protonating the terminal alkene or epoxide of the prenyl diphosphate precursor [13]. Many TPSs can produce more than one product with distinct scaffolds from one substrate, further enhancing structural diversity [14].

Following the formation of sesquiterpenoid carbon skeletons, tailoring enzymes catalyze functional modifications that further diversify both structure and bioactivity. Cytochrome P450 monooxygenases (P450s) are the most prominent enzymes catalyzing various reactions, including the hydroxylation of deactivated C–H bonds [15], olefin epoxidation [16], demethylation [17], and multi-electron oxidations [18]. These modifications increase polarity and reactivity, often conferring novel bioactivities. Other oxidoreductases, such as non-heme Fe/α-ketoglutarate-dependent oxygenases [19], flavin-dependent monooxygenases [20], and short-chain dehydrogenases/reductases [21], are also common enzymes in terpenoid biosynthetic pathways, contributing to scaffold elaboration via oxidative transformations. Some transferases also contribute to the diversity of terpenoid products, including methyltransferases [22], acetyltransferases [23] and glycosyltransferases [24]. Although most tailoring enzymes are highly selective in terms of chemistry, regioselectivity, and stereochemistry, some exhibit substrate or product promiscuity, producing several closely related metabolites in a single pathway, thereby further enriching sesquiterpenoid chemical diversity [25].

Earlier research involving the detection of cryptic clusters, ranging from single-strain metabolome-guided mining [26] to genus-wide pangenome surveys [27], demonstrated the utility of antiSMASH for identifying biosynthetic gene clusters (BGCs) in *Streptomyces* species. The identification and application of strong promoters, such as *ermEp∗* [28], *kasOp∗* [29], and *stnYp* [30], have been crucial for activating silent BGCs and driving transcription leading to metabolite production. Moreover, promoter insertion represents a widely used strategy for BGC activation [31]. Heterologous expression provides another effective approach to activating silent BGCs. Specifically, it involves refactoring the targeted BGCs and introducing them into suitable chassis strains of specific species, such as *Streptomyces albus* [32], *Streptomyces coelicolor* [33], and *Streptomyces lividans* [34]. Among these strains, *S. albus* J1074 has been widely applied for the heterologous expression of diverse natural products-BGCs (NP-BGCs) because of its well-characterized genetic background, fast growth, chemical profile, and efficient genetic manipulation [35]. Previous studies using such chassis strains have identified various novel natural products, including fredericamycin [36], napyradiomycin [37], and moenomycin A [38]. In earlier studies on the activation of NP-BGCs, target BGC transcription levels were typically monitored by RT-qPCR analyses [39], while the desired compounds were detected via high-performance liquid chromatography [40] and mass spectrometry [41]. Various efforts have collectively established an efficient pipeline for identifying natural products.

In this study, a new cryptic *pha*-BGC containing MVA pathway-related genes was identified on the basis of genome mining. By engineering the targeted BGC promoter and comparing functional gene transcription levels determined by RT-qPCR and metabolomic profiles revealed by LC-ESI-MS of the wild-type and derived strains, we identified four new 6/5-fused bicyclic sesquiterpenoid compounds, designated as phaterpene A–D (**1**–**4**). Moreover, the heterologous expression of the TPS gene *phaA* in *E. coli* yielded the cyclized product phaterpene F (**5**), suggesting it is involved in the catalyzed formation of a six-membered ring sesquiterpene skeleton. By cluster refactoring and targeted gene knockout, a novel intermediate metabolite, phaterpene E (**6**), was identified, which further clarified the phaterpene biosynthetic pathway. Thus, this study revealed a new 6,5-fused bicyclic sesquiterpenoid scaffold and elucidated its biosynthetic pathway, while also demonstrating that the combined strategy applied in this study is potentially relevant to the targeted identification of new natural products.

## 2 Materials and methods

### 2.1 Strains, plasmids, and media

Details regarding strains, plasmids, primers, and culture media are provided in Supplementary Table 1–4. OSK-123 was used as a wild-type strain containing *pha*-BGC, whereas *S. albus* J1074 served as the heterologous host for expressing this gene cluster. FX-1 and FX-19 were used for phaterpene A–D (**1**–**4**) production, while FX-20, FX-21, FX-22, FX-23, and FX-24 were used to analyze phaterpene biosynthesis. Additionally, FX-21 was used for phaterpene E (**6**) production. *E. coli* strains DH10B, ET12567 (pUZ8002), GB08-red, and BL21 (DE3) served as hosts for gene cloning, conjugation, gene knockout, and protein expression, respectively. Episomal vector pJTU1278 and its derivative plasmids were used for promoter replacement. Integrative vector pSET152 and its derivative plasmids were used for heterologous expression and gene functional analyses. Vector pET21a and its derivative plasmids, including pMH1 and pFZ81, were used to functionally characterize proteins. FX-30 was used for phaterpene F (**5**) production.

### 2.2 Isolation and 16S rDNA identification of strain OSK-123

Strain OSK-123 was isolated from soil on Dongshan Island, Fujian, China (23°44′59″ N, 117°30′4″ E) in 2022. Soil samples were collected in paper bags and stored in darkness. The collected soil was mixed with sterile water and then spread on the surface of ISP2 agar medium supplemented with nystatin (50 μg/mL; inhibitor of fungal growth) and nalidixic acid (50 μg/mL; inhibitor of Gram-negative bacterial growth) in plates. Culture plates were incubated at 30 °C for 7 days and OSK-123 was purified via continuous passage. The isolated strain was stored in 20 % glycerol (v/v) at −80 °C.

Genomic DNA was extracted from strain OSK-123 using the GENEray™ Bacterial Genomic DNA Extraction Kit according to the manufacturer's protocol and was used as the template for PCR amplification with primers 27F and 1492R. Amplified products were sequenced and then the resulting sequences were compared against sequences in the GenBank database.

### 2.3 Genome sequencing and bioinformatic analysis

TSBY liquid medium was inoculated with OSK-123 spores and incubated at 30 °C with shaking (220 rpm) for 2 days. The resulting bacterial cells were collected and sent to Shanghai Personalbio technology CO., LTD for whole-genome sequencing, which was performed using both Illumina NovaSeq (second-generation) and Oxford Nanopore ONT (third-generation) platforms. The obtained reads were assembled using HGAP v4 and CANU v1.7.1 to generate a complete genome sequence. The complete genome sequence has been submitted to the National Microbiology Data Center (https://nmdc.cn/resource/genomics/sequence/detail/NMDCN0005RCV). The following online tools and databases were used in this study: secondary metabolite prediction tool antiSMASH bacterial version 8.0.1 (https://antismash.secondarymetabolites.org/); biosynthetic gene cluster family database BiG-FAM (https://bigfam.bioinformatics.nl/); EFI-Enzyme similarity tool and sequence similarity network (SSN) version 2025_02/105 (https://efi.igb.illinois.edu/efi-est/); and minimum information about a biosynthetic gene cluster (MIBiG) version 4.0 (https://mibig.secondarymetabolites.org/).

### 2.4 Fermentation of strain OSK-123 and *S*. *albus* J1074

Bacteria were maintained on SFM agar medium in plates for 4–5 days. A 2 cm^2^ agar plug containing bacterial cells was used to prepare a 30 mL TSBY seed culture, which was incubated at 30 °C with shaking (220 rpm) for 2 days. Next, 10 % of the seed culture was transferred to 30 mL R5 fermentation medium and cultured at 30 °C with shaking (220 rpm) for 7 days. For large-scale fermentation, 20 mL seed culture was spread evenly on 1 L R5 agar medium in a sterilized stainless steel plate and allowed to dry on a clean bench. Mutants (e.g., FX-1 and FX-21) were incubated at 30 °C for 7 days.

### 2.5 Gene cluster promoter replacement in strain OSK-123

To replace the *pha*-BGC promoter, homologous flanking sequences (1-L and 1-R) were amplified by PCR using the corresponding primers designed for the strain OSK-123 genome and then cloned into the pJTU1278 vector. Subsequently, a DNA fragment containing strong promoters (*stnYp* and *kasOp∗*) and the apramycin resistance gene *aac(3)IV* was inserted into the vector to construct pLQ1501, which was verified by sequencing. *E. coli* ET12567/pUZ8002 cells were transformed with the recombinant plasmid, which was then introduced into strain OSK-123 via intergeneric conjugation. After two rounds of growth on non-selective SFM medium in plates, the mutant strain resistant to apramycin was cultured on SFM agar medium in plates and verified by PCR.

### 2.6 Intergeneric conjugation between *E. coli* and OSK-123 or *S. albus* J1074

*E. coli* ET12567/pUZ8002 cells containing recombinant plasmids were cultured overnight at 37 °C in LB medium supplemented with 50 mg/L apramycin, 25 mg/L chloramphenicol, and 50 mg/L kanamycin. The overnight culture was used to inoculate (1/100, v/v) fresh LB medium, which was then incubated until the optical density at 600 nm (OD_600_) reached 0.4–0.6. Cells were washed three times with fresh LB medium and resuspended in the same volume of LB medium. Strain OSK-123 was grown on SFM medium in a plate for 8–10 days. Spores were collected using cotton swabs and then suspended in 0.05 mol/L TES buffer. They were subsequently filtered through sterile cotton to remove mycelia and then washed three times with TES buffer before being resuspended in 2 × YT medium. Following a 10-min heat shock treatment at 50 °C, 1 μL 5 mol/L CaCl_2_ was added and then the mixture was incubated at 37 °C for 2.5 h. Next, 0.5 mL spores and 0.5 mL *E. coli* cells were mixed and spread on SFM agar medium containing 20 mM MgCl_2_ in plates, which were incubated at 30 °C for 16–20 h before 0.8 mL sterile water containing 1 mg apramycin and 1 mg trimethoprim was spread over the medium surface. Plates were incubated at 30 °C for another 5–7 days.

### 2.7 Heterologous expression of the *pha* gene cluster in *S*. *albus* J1074

For the heterologous expression of the *pha* gene cluster, a PCR amplification was performed and pSET152 was used to generate recombinant plasmid pLQ1512 carrying the *pha* gene cluster along with the strong promoters *stnYp* and *kasOp∗*. The subsequent intergeneric conjugation was performed using the same procedure as that used for OSK-123. The positive mutant resistant to apramycin was cultured on SFM agar medium in a plate and verified by PCR.

### 2.8 HPLC and LC-HRMS analysis of the related compounds

Relative abundances of natural products were determined by HPLC (Agilent series 1260, Agilent Technologies, USA), with detection wavelengths of 210 or 250 nm. Molecular weights were analyzed using an HPLC-time-of-flight mass spectrometry (HPLC-TOF/MS) system (Agilent 1290–6230 TOF); the same HPLC method was used, with detection in the positive ion mode. HPLC and LC-MS analyses were conducted using the following: column, Agilent Eclipse XDB-C18 column (4.6 × 250 mm, 5 μm); flow rate, 0.5 mL/min; injection volume, 10 μL; linear gradient, H_2_O: MeOH = 90 : 10, reached H_2_O: MeOH = 0 : 100 at 30 min and held until 35.0 min before returning to H_2_O: MeOH = 90 : 10 at 35.01 min and equilibrating for 5.0–40 min.

### 2.9 Extraction and purification of phaterpene A–E

Large-scale fermentation products (20 L) of mutant FX-1 or FX-21 was cut into 2 cm^2^ pieces for an extraction (six times with methanol). Water was added to the solid residue before a second extraction (six times with ethyl acetate) was completed. All extracts were combined and evaporated under vacuum conditions using a rotary evaporator. The obtained crude fermentation products were initially separated using MCI GEL CHP20 as well as 30 %, 50 %, 70 %, and 100 % methanol (1 L each). Related products were detected by HPLC. Collected samples were then purified using Sephadex LH-20 with an appropriate MeOH: H_2_O ratio. Related products were detected by HPLC. Finally, preparative liquid chromatography (column: Agilent Eclipse XDB-C18, 9.4 × 250 mm, 5 μm) was used to separate products until purity exceeded 90 %. Structural information about relevant compounds was obtained via nuclear magnetic resonance (NMR). Spectra were acquired using a 600 MHz Bruker Avance III NMR spectrometer (^1^H: 600 MHz; ^13^C: 150 MHz). Chemical shifts were determined in terms of ppm and relative to the signal of the residual solvent CDCl_3_ (^1^H: 7.26 ppm; ^13^C: 77.16 ppm). Coupling constants (*J*) were determined in terms of Hz and peak types were categorized as singlet (s), doublet (d), doublet of doublets (dd), multiplet (m), and broad (br) (Fig. 3a).

### 2.10 Gene knockout using Red/ET recombination in *E. coli* GB08-red

A DNA fragment containing the ampicillin resistance gene (*amp*^*R*^) and 20–50 bp of its flanking sequences was amplified by PCR. The amplified DNA fragment was purified and introduced into *E. coli* GB08-red/pLQ1512 cells via electroporation (1.4 kV, 10 μF, and 600 Ω) after cells were induced with 50 μL 10 % l-arabinose for 35 min at 37 °C. Following the addition of 600 μL LB medium, the sample was incubated at 37 °C with shaking (220 rpm) for approximately 90 min. Cultures were added to LB agar medium containing ampicillin (50 μg/mL), apramycin (50 μg/mL), and kanamycin (50 μg/mL) in plates. Clones were grown on LB agar medium in plates and verified by PCR and sequencing.

### 2.11 RT-qPCR analysis

For an RT-qPCR analysis of *Streptomyces*, mycelia were harvested from a 1 mL fermentation culture via centrifugation (12,000 rpm for 1 min at 4 °C). The collected mycelia were ground for 5 min using liquid nitrogen and a mortar, after which the ground material was resuspended with 0.5 mL Redzol. Total RNA was extracted from samples using an Quazol™ RNA extraction reagent (ShareBio). RNA quality and concentration were determined using a NanoDrop One spectrophotometer (Thermo Fisher). Residual genomic DNA was digested by DNase I (All-in-One First-Strand Synthesis MasterMix, ShareBio) and then the digested sample was used as a template for a PCR amplification using 123*hrdB*-RT-F/123*hrdB*-RT-R or 1074*hrdB*-RT-F/1074*hrdB*-RT-R primer pairs to verify whether DNA was completely removed. The remaining RNA was reverse transcribed to synthesize cDNA using All-in-One First-Strand Synthesis Mastermix (All-in-One First-Strand Synthesis MasterMix, ShareBio). Transcription of the related genes was monitored by RT-qPCR using analytikjena qTOWER3G touch with a Maxima™ SYBR Green/ROX qPCR kit (Thermo Fisher). Relative transcription levels were calculated according to the 2^−ΔΔCt^ method, with the housekeeping gene *hrdB* serving as an internal control.

### 2.12 Functional analysis of the TPS (PhaA)

To functionally characterize PhaA, engineered *E. coli* FX-30 cells were cultured at 37 °C in 500 mL flasks containing 150 mL fermentation medium supplemented with 50 μg/mL ampicillin, 50 μg/mL kanamycin, and 50 μg/mL chloramphenicol. When OD_600_ reached 0.6–0.8, IPTG was added for a final concentration of 0.4 mM. After a 2-day incubation at 30 °C, 10 mL fermentation broth was collected for an overnight extraction using 20 mL ethyl acetate. The organic layer was concentrated using a rotary evaporator and dissolved with 1 mL methanol. Fermentation products were detected by HPLC and LC-MS.

### 2.13 Quantum chemical calculations

Conformational analysis was performed using the GMMX module in GaussView with the MMFF94 force field, retaining conformers within a 3.5 kcal/mol energy threshold. All selected conformers were subsequently optimized at the DFT/B3LYP/6-311+G(d,p) level using Gaussian 16. The ECD spectra were calculated using time-dependent DFT (TD-DFT) at the CAM-B3LYP/6-311+G(2d,p) level with methanol as the solvent, while the NMR parameters were computed using the GIAO method at the MPW1PW91/6-311+G(2d,p) level using the IEFPCM solvation model in chloroform. The final calculated values for both ECD and NMR were derived from a Boltzmann average of the contributing conformers. The computed ^13^C NMR chemical shifts were then subjected to DP4+ analysis to quantitatively assess the probability of the proposed structure.

## 3 Results

### 3.1 The *pha* cluster putatively encodes the new sesquiterpenoid compounds

**Fig. 1:**
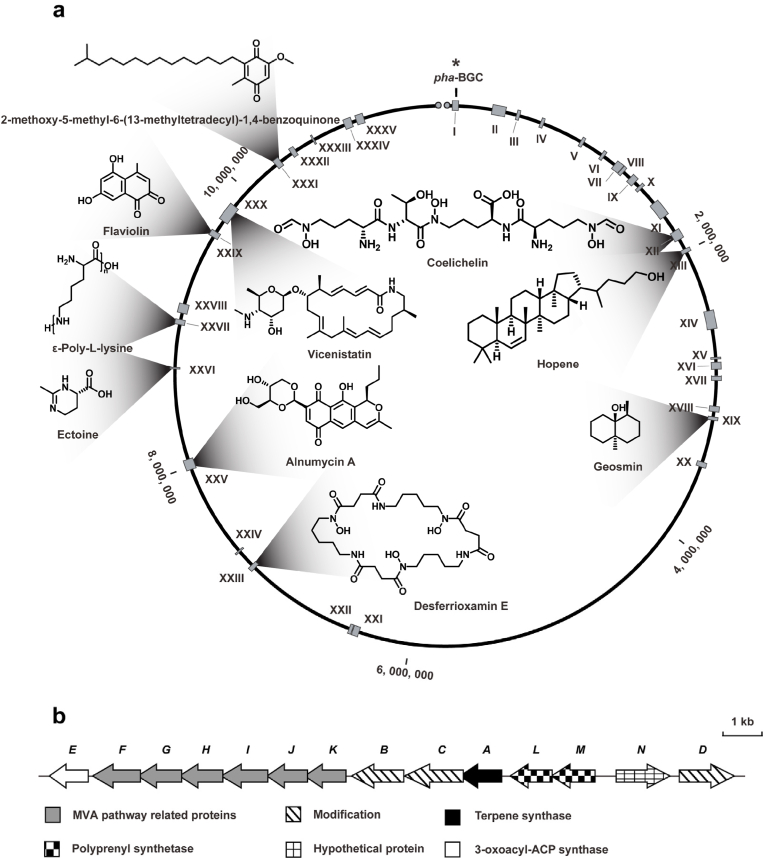
**Genome mining resulted in the discovery of new *pha* cluster.** a, Analysis of secondary metabolite biosynthetic gene clusters in strain OSK-123. b, *pha* biosynthetic gene cluster in strain OSK-123.

Strain OSK-123 was isolated from a soil sample and identified as *S. phaeochromogenes* according to the results of a 16S rDNA sequence similarity analysis. Whole-genome sequencing data revealed its genome consists of 11.5 Mbp and includes 9,833 predicted genes. The secondary metabolite prediction software antiSMASH bacterial version 8.0.1 [42] identified approximately 35 putative BGCs in this strain, approximately two-thirds of which were predicted to be associated with unknown products (Fig. 1a and Supplementary Table 5). Specifically, a terpenoid-related cluster harboring an MVA pathway gene, which was designated as *pha*-BGC, spanned an approximately 18 kb DNA region containing 14 open reading frames (Fig. 1b). A detailed analysis of the similarity between *pha*-BGC and sequences in the BiG-FAM database [43] combined with a cluster visualization [44] indicated that, with the exception of the gene cluster itself in other *Streptomyces* species, there were no highly similar gene clusters, suggesting that *pha*-BGC encodes proteins associated with new compounds (Supplementary Fig. 1). A functional annotation of *pha*-BGC genes indicated that they likely encode the following: PhaA, TPS; PhaB and PhaC, protoporphyrinogen/coproporphyrinogen oxidases; PhaD, cytochrome P450; PhaL and PhaM, polyprenyl synthetases; PhaF–PhaK, MVA pathway proteins; PhaN, hypothetical protein; PhaE, 3-oxoacyl-ACP synthase (Supplementary Table 6). A comparative analysis of selected protein sequences (PhaA, PhaB, and PhaC) and proteins in the MIBiG database [45] revealed low sequence similarity (Supplementary Table 7). According to an SSN analysis [46,47], PhaA formed a small, isolated cluster along with two other proteins. Similarly, the biosynthetic enzymes PhaB and PhaC were uniquely clustered (Supplementary Fig. 2). NP-BGCs containing genes encoding these proteins remain uncharacterized. These findings suggest that these functional genes might be responsible for the biosynthesis of new sesquiterpenes. Hence, on the basis of a comparative analysis of the complete gene cluster and key biosynthetic genes, we determined that *pha*-BGC encodes proteins associated with novel compounds of interest.

### 3.2 Strong promoter replacement awakened *pha*-BGC expression

**Fig. 2:**
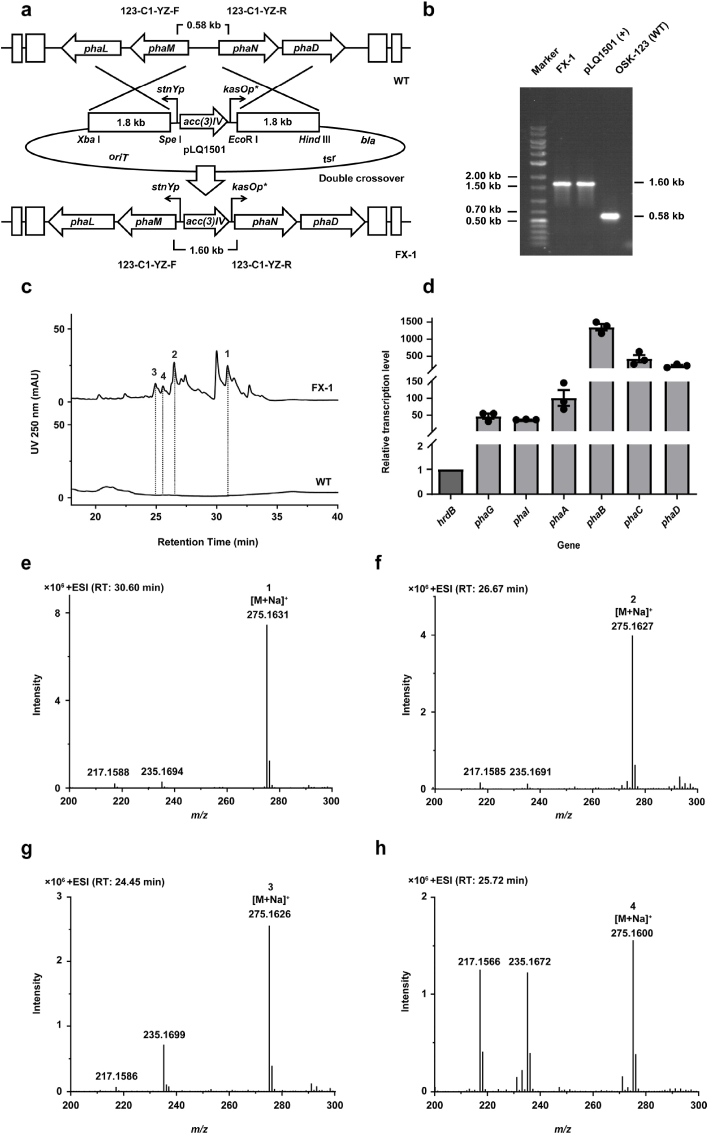
**New metabolites were identified through promoter replacement in strain OSK-123.** a, Schematic diagram of the construction of mutant strain FX-1. b, Verification of FX-1. c, HPLC profiles of fermentation products of FX-1 and OSK-123 (250 nm). d, Relative transcription levels of *phaG*, *phaI*, *phaA*, *phaB*, *phaC*, and *phaD* in FX-1. e, HRMS analysis of phaterpene A (**1**). f, HRMS analysis of phaterpene B (**2**). g, HRMS analysis of phaterpene C (**3**). h, HRMS analysis of phaterpene D (**4**).

The lack of information regarding the chemical structures of compounds associated with *pha*-BGC genes made it difficult to detect and identify target compounds in OSK-123 cultures via chromatography or spectroscopy. A promoter replacement experiment was conducted to enhance *pha*-BGC functional gene transcription, thereby increasing the production of target products in the OSK-123 culture broth. More specifically, the strong promoters *stnYp* and *kasOp∗* were added to the intergenic region between *phaM* and *phaN* via homologous recombination. Of these promoters, *stnYp* was located upstream of *phaM* and controlled the transcription of *phaM* and the downstream genes. By contrast, *kasOp∗* was located upstream of *phaN* and controlled the transcription of *phaN* and the downstream genes. Moreover, *acc(3)IV* was inserted into the junction between *stnYp* and *kasOp∗* to facilitate the screening of derivative strains during conjugation experiments. The resulting mutant was verified by PCR (1.60 kb product) and named FX-1. The corresponding PCR amplification for the wild-type strain OSK-123 generated a 0.58 kb product (Fig. 2a and b).

**Fig. 3:**
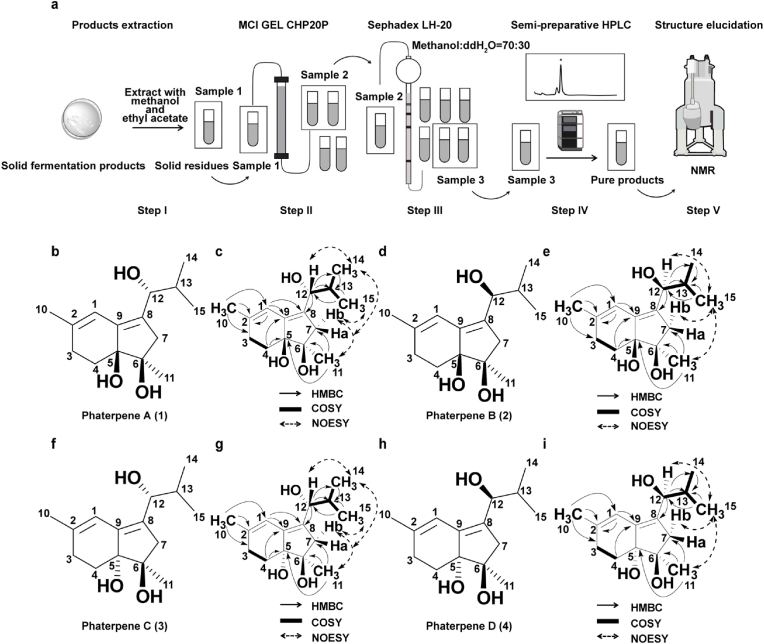
**Isolation and structural elucidation of phaterpenes A–D (compounds 1**–**4).** a, Workflow diagram for natural product purification. The crude product (Sample 1) was extracted from the fermentation medium using methanol and ethyl acetate (Step I). Sample 1 was separated according to polarity using MCI GEL CHP20, with fractions containing target compounds combined to yield Sample 2 (Step II). Sample 2 was then separated according to molecular weight using a Sephadex LH-20 column, with fractions containing target compounds combined to obtain Sample 3 (Step III). Target compounds were purified via semi-preparative liquid chromatography to obtain the final product (Step IV). Finally, structural information for the final product was obtained via NMR (Step V). b, Chemical structure of phaterpene A (**1**). c, HMBC, COSY, and NOESY details for phaterpene A (**1**). d, Chemical structure of phaterpene B (**2**). e, HMBC, COSY, and NOESY details for phaterpene B (**2**). f, Chemical structure of phaterpene C (**3**). g, HMBC, COSY, and NOESY details for phaterpene C (**3**). h, Chemical structure of phaterpene D (**4**). i, HMBC, COSY, and NOESY details for phaterpene D (**4**).

The fermentation extracts of FX-1 and OSK-123 were compared. Both strains were cultured in 10 different liquid media (Supplementary Table 4) for 7 days at 30 °C with shaking (220 rpm). After fermentation, the crude extract was obtained and analyzed using an XDB-C18 chromatographic column (Supplementary Fig. 4a and 4b). For the R5 medium fermentation, a series of peaks was clearly observed at a retention time spanning 20–35 min for mutant strain FX-1, but not for strain OSK-123 (Fig. 2c). In LC-ESI-MS profiles, the molecular weight of all peaks was 252.1725, suggesting that the detected compounds were present as isomers (Fig. 2e, 2f, 2g and 2h). To determine the transcription levels of *pha*-BGC functional genes during the R5 medium fermentation, a comparative RT-qPCR analysis of FX-1 and OSK-123 was performed. Several genes in the MVA pathway as well as the TPS gene *phaA* and modification genes *phaB*, *phaC*, and *phaD* were selected for the RT-qPCR analysis. The transcription levels of these genes were significantly higher in FX-1 than in OSK-123 (Fig. 2d), which was consistent with the LC-ESI-MS results. Notably, key biosynthetic genes were transcribed at very low levels in OSK-123, suggesting that *pha*-BGC is silent under standard laboratory conditions. Thus, combining a comparative analysis of different fermentation extracts of OSK-123 and FX-1 via LC-ESI-MS with RT-qPCR was useful for detecting novel compounds.

### 3.3 Isolation and structural elucidation of new phaterpenes A–D

Almost all of the peaks detected by LC-ESI-MS had the same molecular weight (252.1725), implying that the compounds are a series of isomers, making them relatively difficult to purify. To elucidate the chemical structures of the new compounds, 20 L R5 agar medium cultures of strain FX-1 were prepared. After fermentation, the R5 agar medium in plates was harvested for an extraction using methanol. Crude extracts were partitioned using ethyl acetate and then the organic layer was concentrated under vacuum conditions to yield the final extract. The prepared sample was then analyzed using MCI GEL CHP20, Sephadex LH-20, and preparative liquid chromatography to isolate purified compounds **1** (28.1 mg), **2** (23.5 mg), **3** (5.3 mg) and **4** (3.2 mg). Finally, their chemical structures were elucidated on the basis of 1D and 2D NMR spectroscopy and HRMS (Fig. 3a).

Compound **1** was isolated as a white solid powder. Its molecular formula C_15_H_24_O_3_ was deduced from HR-ESI-MS data: *m/z* 275.1631 ([M + Na]^+^, C_15_H_24_O_3_Na^+^, calculated value 275.1618), indicating four degrees of unsaturation. The characteristic UV absorption wavelength is presented in Supplementary Fig. 18a. The ^1^H NMR spectrum (600 MHz, *CDCl*_*3*_) of **1** included four methyl group signals [1.78 (s), 1.14 (s), 0.96 (d, *J =* 5.17), and 0.67 (d, *J =* 5.45)], one olefinic proton signal [6.01 (s)], and one hydroxyl proton signal [4.01 (d, *J =* 8.75 Hz)], along with other proton signals consistent with the structure. The ^13^C NMR spectrum (150 MHz, *CDCl*_*3*_) included 15 carbon signals, with four methyl group signals [22.94, (C-11); 22.51, (C-10); 17.97, (C-14); and 17.79, (C-15)], three methylene group signals [44.12, (C-7); 27.07, (C-3); and 25.38, (C-4)], one methine group signal [31.16, (C-13)], four olefinic carbon signals [139.32, (C-2); 135.40, (C-9); 135.20, (C-8); and 115.01, (C-1)], and three oxygenated carbon signals [79.46, (C-5); 76.87, (C-6); and 72.51, (C-12)], which are characteristics of a sesquiterpene skeleton. In the HMBC spectrum, correlations between H-1 and C-2, C-3, C-5, C-9, and C-10 as well as between H-4a and C-2, C-3, C-5, C-6, and C-9 reflected the presence of a hexacycle fused to a pentacycle skeleton. The partial structure of an isobutyl alcohol group at C-8 was established by HMBC (H-9 and H-10 correlated with C8) and ^1^H–^1^H COSY (H-10 correlated with H-9, H-14, and H15). According to NOESY analysis, three correlations (H-12 with H-15, H-7a with H-15, and H-7b with H-11) provided the information that the signals of protons at H-12, H-15 and H-7a located at the same orientation, while H-11, H-7b and H-14 were situated at another orientation (Fig. 3c). Accordingly, on the basis of the 1D and 2D NMR spectra of its chemical structure (Supplementary Fig. 12 and Supplementary Table 9), **1** was identified as a new compound and named phaterpene A (**1**) (Fig. 3b).

Compound **2** was similar to compound **1** in terms of physical and spectral characteristics. It also had the same molecular formula (C_15_H_24_O_3_) according to HR-ESI-MS data: *m/z* 275.1631 ([M + Na]^+^, C_15_H_24_O_3_Na^+^, calculated value 275.1618). The characteristic UV absorption wavelength is presented in Supplementary Fig. 18b. The 1D and 2D NMR spectra of **2** were similar to those of **1**, but HPLC profiles revealed compounds **1** and **2** had clearly different retention times. Hence, compounds **1** and **2** were likely isomers. Correlation analyses of HMBC and ^1^H–^1^H COSY data suggested compound **2** shares the same planar molecular skeleton as compound **1**. However, on the basis of NOESY analysis, the correlations of H-12 with H-14, H-7b with H-11, and H-7b with H-14 suggested that the protons at H-12, H-7b, H-11 and H-14 were located at the same orientation, suggesting the different relative configuration of C-6 and C-12 between compounds **2** and **1** (Fig. 3e) (Supplementary Fig. 13 and Supplementary Table 10). After determining its chemical structure, compound **2** was named phaterpene B (**2**) (Fig. 3d).

Furthermore, based on the HR-ESI-MS analysis, both of compound **3** and **4** shared the identical molecular formula C_15_H_24_O_3_ and UV absorptions (Supplementary Fig. 18c and d). The 1D and 2D-NMR data of **3** (Fig. 3f and g, Supplementary Fig. 14 and Supplementary Table 11) and **4** (Fig. 3h and i, Supplementary Fig. 15 and Supplementary Table 12) are quite similar to those of compounds **1** and **2**. The 2D-NMR correlations of HMBC and ^1^H,^1^H COSY revealed that the identical planar structures of compounds **3**–**4** were consistent with those of compounds **1**–**2**. Further NOESY analysis displayed that compounds **3** and **4** were the C-12 diastereomer pairs as well. Thus, the chemical structures of compounds **3**–**4** were assigned as phaterpene C (**3**) and phaterpene D (**4**) and shown as in Fig. 3f to i.

The configuration of phaterpene A–D (**1**–**4**) was determined via a combined approach of ^13^C NMR calculation with DP4+ analysis [[48], [49]] and electronic circular dichroism (ECD) simulation, a robust strategy for chiral natural product structural elucidation. For relative configuration screening, four proposed configurations (5*S*∗6*R*∗12*R*∗, 5*S*∗6*R*∗12*S*∗, 5*R*∗6*R*∗12*S*∗, 5*R*∗6*R*∗12*R*∗) were subjected to ^13^C NMR calculations. DP4+ analysis clearly assigned compounds **1**/**2** to the 5*S*∗6*R*∗ scaffold (DP4+ values > 99 %) and compounds **3**/**4** to the 5*R*∗6*R*∗ scaffold (DP4+ values > 99 %). Notably, the C-12 chiral center-located on a flexible side chain-exerted negligible effects on ^13^C NMR shifts, rendering it indistinguishable via NMR, thus confirming compounds **1**/**2** and **3**/**4** as C-12 diastereomer pairs (Supplementary Table 15). The absolute configurations were further validated by ECD simulations. The theoretical spectra showed good agreement with the experimental CD data, confirming the 5*S*, 6*R* configuration for compounds **1**/**2** and the 5*R*, 6*R* configuration for compounds **3**/**4** (Supplementary Fig. 20). Combination with the NOSEY correlations in compound **1**–**4**, the corresponding *R* or *S* configurations of C-12 were further established. In a word, this study underscored the efficacy strategy through integration of NOESY, NMR-DP4+ and ECD for structural elucidation of similar scaffolds.

### 3.4 PhaA catalyzed the formation of a six-membered ring sesquiterpene skeleton

**Fig. 4:**
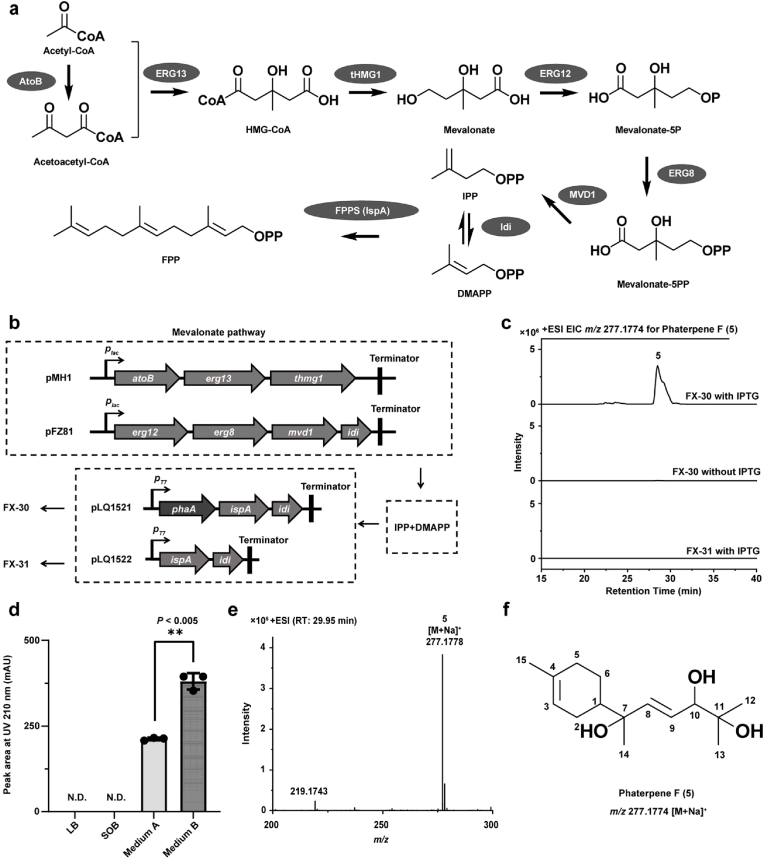
**Functional validation of the terpene synthase PhaA.** a, Proteins involved in FPP production in *E. coli*. AtoB, acetyl-CoA acetyltransferase; ERG13, HMG-CoA synthase; tHMG1, truncated 3-hydroxy-3-methylglutaryl-CoA reductase; ERG12, mevalonate kinase; ERG8, phosphomevalonate kinase; MVD1, mevalonate pyrophosphate decarboxylase; Idi, IPP isomerase; FPPS, FPP synthase; IspA, FPPS from *E. coli*; HMG-CoA, (*S*)-3-hydroxy-3-methylglutaryl-CoA; DMAPP, dimethylallyl pyrophosphate; IPP, isopentenyl diphosphate; FPP, farnesyl pyrophosphate. b, Construction of mutants FX-30 and FX-31. c, HRMS-based identification of target compounds from fermentation cultures of strain FX-30 induced with IPTG and comparison with the fermentation products of FX-30 that was not induced with IPTG and the fermentation products of FX-31 induced with IPTG. d, Optimization of the culture medium for FX-30; *P* values were calculated using a two-tailed Student's *t*-test assuming unequal variance; ∗, *P <* 0.01; ∗∗, *P <* 0.005; ∗∗∗, *P <* 0.001. e, HRMS analysis of phaterpene F (**5**). f, Chemical structure of phaterpene F (**5**).

To further explore the basic structural characteristics of sesquiterpenes, a biosynthetic pathway involving the precursor and TPS encoded by *phaA* was reconstructed in *E. coli* cells (Fig. 4a). TPSs typically use FPP as a substrate to generate sesquiterpene products. FPP was formed from two molecules of IPP and one molecule of DMAPP in a reaction catalyzed by IspA, which is an FPP synthase. Plasmids pMH1 and pFZ81 containing all of the functional genes responsible for IPP and DMAPP biosynthesis were constructed in a previous study [50]. Considering *idi* is associated with a bottleneck during the biosynthesis of FPP, we constructed pLQ1521 by cloning the codon-optimized *phaA*, *ispA*, and *idi* operon into pET21a under the control of the T7 promoter (Fig. 4b). Additionally, to produce soluble proteins in *E. coli* cells, *phaA* codons were optimized, which decreased the GC content from 71.15 % to 58.09 %, whereas the codon adaptation index increased from 0.47 to 0.74 (Supplementary Fig. 11). A control plasmid (pLQ1522) was also constructed by cloning only the *ispA* and *idi* operon into pET21a under the control of the T7 promoter (Fig. 4b).

*E. coli* BL21 (DE3) cells were transformed with pMH1, pFZ81, and pLQ1521 via electroporation to generate strain FX-30. Similarly, *E. coli* BL21 (DE3) cells were transformed with pMH1, pFZ81, and pLQ1522 via electroporation to obtain control strain FX-31 (Fig. 4b). Strains FX-30 and FX-31 were cultured in Medium A (Supplementary Table 4) reported in a previous study [50] and induced with IPTG. The fermentation broth extract obtained using ethyl acetate was analyzed by HPLC. A new peak for a low-abundant compound (**5**) was detected at 29.95 min for FX-30 induced with IPTG, but not for FX-31 or FX-30 that was not induced with IPTG (Fig. 4c). Moreover, according to LC-HR-ESI-MS data, the target compound had a *m/z* value of 277.1778 [M + Na]^+^ and a predicted molecular formula of C_15_H_26_O_3_ (Fig. 4e). These findings suggest that compound **5** may be the final product of the reconstructed sesquiterpene biosynthetic pathway.

The low abundance of compound **5** in the Medium A of strain FX-30 made it difficult to purify and structurally characterize this compound. Hence, we optimized the medium composition to increase the production of compound **5**. Four fermentation media were evaluated, including LB, SOB, Medium A, and Medium B (Supplementary Table 4), which were supplemented with IPTG. Of these media, Medium B resulted in the highest production of compound **5** (79.37 % higher than in Medium A) (Fig. 4d). Therefore, strain FX-30 was cultured in 10 L Medium B and induced with IPTG. Culture extracts were then prepared and separated using different chromatographic techniques to purify compound **5** (12.4 mg).

The chemical structure of compound **5** was determined using spectroscopy-related methods, including 1D and 2D NMR and HR-ESI-MS analyses (Supplementary Fig. 16 and Supplementary Table 13). The molecular formula of compound **5** was determined (*m/z* 277.1778) through an HR-ESI-MS analysis ([M + Na]^+^, C_15_H_26_O_3_Na^+^, calculated value 277.1774), with three degrees of unsaturation. The characteristic UV absorption wavelength is presented in Supplementary Fig. 18e.The ^1^H NMR spectrum contained three olefinic proton signals [5.79 (d, *J* = 15.68 Hz), 5.66 (dd, *J* = 6.91, 15.67 Hz), and 5.30 (br, s)], a hydroxyl proton signal [3.89 (d, *J* = 11.48 Hz)], four methyl group signals [1.57 (s), 1.20 (s), 1.16 (s), and 1.09 (s)], and other proton signals consistent with the expected structure. The ^13^C NMR spectrum included 15 carbon signals, four olefinic carbon signals [139.99 (C-8), 133.99 (C-4), 126.27 (C-9), and 120.36 (C-3)], and three oxygenated carbon signals [79.31, (C-10); 74.73, (C-7); and 72.90, (C-11)]. HMBC revealed correlations between H-1 and C-2, C-5, C-6, C-7, C-8, and C-14 as well as between H-15 and C-3, C-4, and C-5, reflecting a molecular skeleton comprising a six-membered ring. A combined analysis of HMBC and ^1^H,^1^H COSY data detected the substitution of the branched fatty diol group. The chemical structure of compound **5** was eventually established (Fig. 4f). According to the observed structural characteristics, PhaA catalyzes a reaction involving FPP to produce a sesquiterpene with a six-membered ring skeleton. Moreover, the hydroxylation occurred at C-7, C-10 and C-11 probably due to the modifications of the unpredictable proteins from *E. coli*.

### 3.5 PhaB catalyzed the formation of a 6,5-fused bicyclic sesquiterpenoid scaffold

**Fig. 5:**
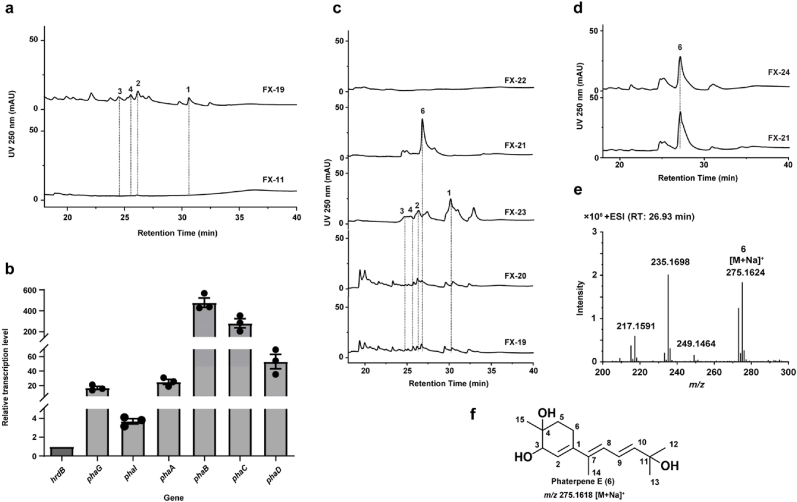
**Identification and structural elucidation of phaterpene E (6).** a, HPLC profiles of fermentation products of FX-11 (*S. albus* J1074::pSET152) and FX-19 (*S. albus* J1074::pLQ1512) (250 nm). b, Relative transcription levels of *phaG*, *phaI*, *phaA*, *phaB*, *phaC*, and *phaD* in FX-19. c, HPLC profiles of fermentation products of FX-19 (*S. albus* J1074::pLQ1512), FX-20 (FX-19Δ*phaN*), FX-21 (FX-19Δ*phaB*), FX-22 (FX-19Δ*phaC*), and FX-23 (FX-19Δ*phaD*) (250 nm). d, HPLC profiles of fermentation products of FX-21 (FX-19Δ*phaB*) and FX-24 (*S. albus* J1074::*phaA* + *phaC*). e, HRMS analysis of phaterpene E (**6**). f, Chemical structure of phaterpene E (**6**).

Notably, *pha*-BGC contained two protoporphyrinogen/coproporphyrinogen oxidase genes (*phaB* and *phaC*) and a cytochrome P450 gene (*phaD*); these genes may encode proteins that contribute to modifications during the biosynthesis of phaterpenes. Therefore, targeted gene inactivation, combined with analysis of changes in mutant fermentation products, enables functional inference of the related gene. However, the inefficient homologous recombination of strain OSK-123 resulted in a time-consuming and labor-intensive analysis of gene functions. To generate an efficient system for elucidating target gene functions, the complete gene cluster was cloned into the *φ*C31 integrative vector pSET152 to generate pLQ1512, in which all functional genes were under the control of a strong promoter (*stnYp* or *kasOp*∗) (Supplementary Fig. 5). Recombinant plasmid pLQ1512 was introduced into *S. albus* J1074 via conjugation to generate the mutant strain FX-19 (*S. albus* J1074::pLQ1512). In addition, the control strain FX-11 carrying the empty vector pSET152 (*S. albus* J1074::pSET152) was generated. Strains FX-19 and FX-11 were cultured in R5 liquid medium. According to an RT-qPCR analysis, *pha*-BGC functional gene transcription levels increased in the mutant (Fig. 5b). After fermentation, HPLC revealed the presence of phaterpene A–D (**1**–**4**) in the extract of strain FX-19 (Fig. 5a). Hence, a phaterpene biosynthetic pathway was reconstructed in the heterologous host *S. albus* J1074. Moreover, the optimized *pha*-BGC may be used to efficiently explore gene functions.

To facilitate genetic manipulations, pLQ1512 was inserted into *E. coli* GB08-red cells, which carry the Red/ET recombination system, for experiments involving linear DNA fragments and circular plasmid recombination. Using pLQ1512 as the basis, *phaN* knockout plasmid pLQ1513 (pLQ1512Δ*phaN*), *phaB* knockout plasmid pLQ1514 (pLQ1512Δ*phaB*), *phaC* knockout plasmid pLQ1515 (pLQ1512Δ*phaC*), and *phaD* knockout plasmid pLQ1516 (pLQ1512Δ*phaD*) were constructed. These plasmids were individually introduced into *S. albus* J1074 via conjugation to generate the following mutants: FX-20 (FX-19Δ*phaN*), FX-21 (FX-19Δ*phaB*), FX-22 (FX-19Δ*phaC*), and FX-23 (FX-19Δ*phaD*) (Supplementary Fig. 6–9). Fermentation extracts for all derivative strains were analyzed by HPLC and LC-ESI-MS. According to the results (Fig. 5c), *phaB*-disrupted strain FX-21 produced compound **6** instead of phaterpene A–D (**1**–**4**). By contrast, *phaC*-disrupted strain FX-22 failed to produce any intermediate related to phaterpene, whereas *phaN*- and *phaD*-disrupted strains FX-20 and FX-23 still produced phaterpene A–D (**1**–**4**), with no significant changes in yield. To further investigate the role of *phaC*, we constructed pLQ1517 carrying the *phaA* and *phaC* operon under the control of the strong promoter *stnYp*. Furthermore, pLQ1517 containing the FPP formation-related genes *phaM* and *phaL* was constructed to increase the production of the final compounds. Mutant strain FX-24 was generated by inserting pLQ1517 into *S. albus* J1074 (Supplementary Fig. 10). A chromatographic analysis detected compound **6** among the fermentation products of FX-24 (Fig. 5d).

To determine the chemical structure of compound **6**, strain FX-21 was cultured on 20 L R5 agar medium, with fermentation extracts prepared for the isolation of highly pure compound **6** (9.8 mg). The characteristic UV absorption wavelength is presented in Supplementary Fig. 18f. Interestingly, ^1^H and ^13^C NMR spectra of compound **6** were similar to those of compound **5**. Moreover, HMBC correlations between H-2 and C-1, C-4, C-6, and C-7 as well as between H-15 and C-3, C-4, and C-5 reflected a six-membered ring with two oxygenated carbon fragments embedded in the molecular skeleton. The substitution of the branched fatty alcohol at C-1 was confirmed through HMBC and ^1^H,^1^H COSY correlations. According to the 1D and 2D NMR spectra and molecular mass of compound **6** (Supplementary Fig. 17 and Supplementary Table 14), compound **6** was structurally characterized and named phaterpene E (Fig. 5e and f). Thus, PhaB specifically mediates the formation of a characteristic five-membered ring in phaterpene A–D (**1**–**4**).

## 4 Discussion

Available genome sequencing methods, bioinformatics tools, and various technologies for silencing NP-BGCs have greatly facilitated the detection and identification of many new bioactive natural products in bacteria for various applications in different fields [51]. Notably, valuable new natural products from actinobacteria, whose chromosomes are inherently rich in NP-BGCs, have been analyzed and various genetic engineering methods have been developed for these strains [52]. However, establishing a direct link between chemical phenotypes and the corresponding NP-BGCs genetic information has been a challenge for the targeted identification of new natural products. AntiSMASH analysis of strain OSK-123 identified a new terpenoid BGC. However, the metabolites derived from this BGC were not detected. The targeted engineering of the *pha-*BGC promoter and a comparison of functional gene transcription levels and LC-ESI-MS profiles between the wild-type strain and mutant strains revealed four new 6/5-fused bicyclic sesquiterpenoid compounds, namely phaterpene A–D (**1**–**4**). Accordingly, combining multiple research strategies is useful for the targeted detection and identification of new natural products.

The intricate metabolic regulatory network in *Streptomyces* controls the expression of NP-BGCs. As a genetically tractable model genus, *Streptomyces* is well-suited for promoter engineering. Native promoters are replaced with a set of strong ones to enhance the transcription of downstream genes and circumvent complex native regulatory mechanisms. This strategy has been used to activate silent BGCs in various *Streptomyces* species and identify a number of new bioactive natural products [53,54]. Moreover, medium composition has critical effects on the physiological metabolism and production of natural products in the engineered strain. Optimizing fermentation conditions and medium composition is a feasible approach to activating silent gene clusters or increasing the yield of target compounds [55]. In the current study, we replaced the *pha*-BGC promoter with strong promoters to enhance the expression of functional genes. By integrating this approach with comparative metabolomic analyses of mutant and wild-type strains grown in different media and RT-qPCR analyses, we rapidly identified a series of target compounds from promoter-optimized strain FX-1. This approach effectively established a link between natural products and related BGCs, facilitating the detection and identification of desired natural products.

**Fig. 6:**
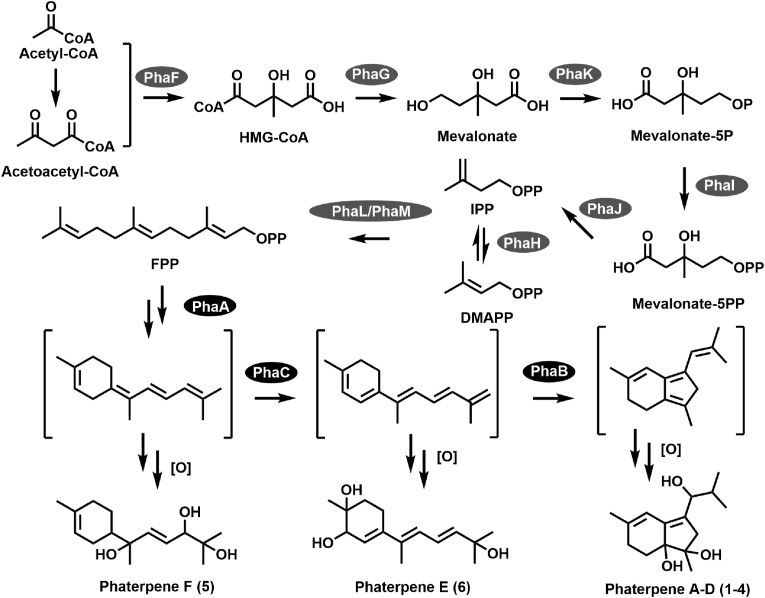
**The putative biosynthesis pathway of phaterpene.** MVA pathway proteins (PhaF–PhaK) are responsible for IPP and DMAPP assembly via acetyl-CoA and acetoacetyl-CoA. The precursor FPP forms in a reaction catalyzed by polyprenyl synthetase (PhaL and PhaM). A terpene synthase (PhaA) mediates the conversion of FPP to a six-membered ring, which is further modified by PhaB and PhaC.

Bacterial protoporphyrinogen oxidases are primarily classified into three types: HemG, HemK, and HemY [56]. An NCBI Conserved Domain Database analysis [57] indicated that both PhaB and PhaC are similar to HemY sequences (COG1232, protoporphyrinogen oxidase HemY/PPOX), with E-values of 1.17e-57 and 2.43e-43, respectively (Supplementary Fig. 19). Therefore, PhaB and PhaC were initially annotated as protoporphyrinogen/coproporphyrinogen oxidases. Protoporphyrinogen/coproporphyrinogen oxidase is an FAD-dependent enzyme that catalyzes the oxidation of protoporphyrinogen-IX to produce protoporphyrin-IX or the oxidation of coproporphyrinogen III to generate coproporphyrin III [58]. Such enzymes play essential roles in the biosynthesis of chlorophyll and heme, which are important for primary metabolic processes, including photosynthesis in plants and aerobic respiration in animals [59]. Sequence similarity analyses also revealed the similarity (38 %) between PhaC and CrtI [60], which is a characterized phytoene desaturase involved in carotenoid biosynthesis, suggesting that these two enzymes may have analogous catalytic functions. PhaB is homologous to SSCG_02152 (41 % similarity) in *Streptomyces clavuligerus* ATCC 27074 [61] and AmcO (31 % similarity) in *Amycolatopsis* sp. AA4 [62], but their functions remain relatively unknown. In this study, targeted gene knockout experiments confirmed that PhaB and PhaC help modify phaterpenes. On the basis of the chemical structures of the intermediates, PhaC may catalyze the desaturation of the substrate to form the conjugated polyene product, while PhaB catalyzes the formation of a five-membered ring. In addition to the functional characterization of a TPS (PhaA) in *E. coli* cells, a biosynthetic pathway for the newly identified phaterpenes was proposed. Specifically, proteins PhaF–K along with PhaL/M are responsible for the biosynthesis of FPP, after which a six-membered ring sesquiterpene skeleton forms in a reaction mediated by the TPS PhaA. Following the desaturation by the protoporphyrinogen oxidase PhaC, another protoporphyrinogen oxidase (PhaB) further mediates the conversion of the five-membered ring to produce the 6,5-fused bicyclic sesquiterpenoid scaffold. Hydroxylations at C-5, C-6, and C-12 result in the production of phaterpene A–D (**1**–**4**) (Fig. 6). Overall, the study findings indicate that protoporphyrinogen oxidases are involved in modifying terpenoids, while also providing insights into a possible phaterpene biosynthetic pathway.

Heterologous expression in a *Streptomyces* chassis strain represents a feasible approach to investigating natural products associated with cryptic BGCs in actinobacteria. Notably, unexpected modifications of target compounds may occur. For example, in a previous study, the heterologous expression of the *epi*-isozizaene-related BGC in *Streptomyces avermitilis* resulted in the P450/epoxidase-mediated oxidation of the desired compound 4,5-epoxy-2-*epi*-zizaan-6β-ol [63]. In another earlier study, cytochrome P450-sb3a in *Sebekia benihana* hydroxylated vitamin D_3_ to produce 25-hydroxyvitamin D_3_ and 1α,25-dihydroxyvitamin D_3_ [64]. CYP260B1 modifies the terpenoid skeleton to generate the oxidized terpenoids culmorin, culmorone and koraidiol [65]. Other unexpected modifications, such as glycosylations [66], halogenations [67], reductions [68], and cluster-crosstalk [69], have also been reported. Although the chemical diversity of products derived from reactions catalyzed by enzymes encoded in *pha*-BGC can complicate metabolite isolation (e.g., dispersed flux and low titers), it does not impede biosynthetic analyses involving knockout or reconstruction strategies. Similarly, for strains in which *pha*-BGC was heterologously expressed and promoter-engineered strains FX-19 and FX-1, we detected diverse products with three hydroxyl group substitutions and differences in stereochemistry. These unexpected hydroxylations might be due to enzymes that are not encoded by *pha*-BGC. The occurrence of hydroxylation substitutions resulting in diastereomers were also observed in other terpenoid products, such as atramacronoid, eudesm-4(15)-ene-7β, 11-diol [70], axinysones [71] and teclenone [72] (Supplementary Fig. 21).

In summary, this study initially detected a new cryptic bacterial terpenoid BGC in strain OSK-123 through an online bioinformatic analysis. To establish the chemical phenotype associated with *pha*-BGC, we integrated several methods to efficiently identify target compounds: promoter engineering, comparative analysis of different fermentation extracts, RT-qPCR confirmation, and LC-ESI-MS detection. Four new 6/5-fused bicyclic sesquiterpenoid compounds, which were named phaterpene A–D (compounds **1**–**4**), were identified. Related functional genes were heterologously expressed in *E. coli* or *Streptomyces* cells. Finally, a phaterpene biosynthetic pathway was proposed. Notably, in this study, an efficient strategy for identifying natural products was developed. Furthermore, the data presented herein may be relevant to further characterizing the chemical structures and biosynthetic mechanisms of new sesquiterpenes.

## CRediT authorship contribution statement

**Xing Fan:** Writing – review & editing, Writing – original draft, Methodology, Investigation, Formal analysis, Data curation, Conceptualization. **Xin Zhang:** Writing – original draft, Formal analysis, Data curation. **Minguo Tang:** Methodology, Formal analysis, Data curation. **Shihao Wei:** Methodology, Formal analysis, Data curation. **Ziyue Guo:** Writing – original draft, Data curation. **Fucai Ren:** Data curation, Formal analysis. **Jiaying Hao:** Formal analysis, Data curation. **Lingqi Hua:** Writing – original draft, Data curation. **Lin Zhou:** Writing – review & editing, Data curation. **Jie Xu:** Writing – review & editing, Data curation. **Wei Huang:** Writing – review & editing, Data curation. **Qianjin Kang:** Writing – review & editing, Writing – original draft, Methodology, Funding acquisition, Data curation. **Linquan Bai:** Writing – review & editing, Supervision, Funding acquisition, Data curation, Conceptualization.

## Declaration of competing interest

The authors declare that they have no known competing financial interests or personal relationships that could have appeared to influence the work reported in this paper. The author Linquan Bai is an Editorial Board Member for *Synthetic and Systems Biotechnology* and was not involved in the editorial review or the decision to publish this article.
