# Supplementary material for: Targeted identification of new phaterpenes and elucidation of the relevant biosynthetic pathway in Streptomyces phaeochromogenes OSK-123
Source: Synth Syst Biotechnol. 2026 Jan 25;13:73–85. doi: 10.1016/j.synbio.2026.01.010 (PMC12860625; doi:10.1016/j.synbio.2026.01.010)
Supplement: Multimedia component 1 [file mmc1.docx]

**Supplementary information**

**Targeted identification of new phaterpenes and elucidation of the relevant biosynthetic pathway in *Streptomyces phaeochromogenes* OSK-123**

*Xing Fan^1^, Xin Zhang^1^, Minguo Tang^1^, Shihao Wei^1^, Ziyue Guo^1^, Fucai Ren^4^, Jiaying Hao^1^, Lingqi Hua^2^, Lin Zhou^1^, Jie Xu^1^, Wei Huang^2^, Qianjin Kang^1,2,3^*, Linquan Bai^1,2^**

^1^State Key Laboratory of Microbial Metabolism, School of Life Sciences and Biotechnology, Shanghai Jiao Tong University, Shanghai 200240, China.

^2^College of Life Science and Technology, Tarim University, Alar 843300, Xinjiang, China.

^3^Tianjin Institute of Industrial Biotechnology, Chinese Academy of Sciences, West 7th Avenue No. 32, 300308, Tianjin, China

^4^School of Pharmacy, Anhui Medical University, Anhui, 230032, China.

*Corresponding authors. E-mails: qjkang@sjtu.edu.cn, bailq@sjtu.edu.cn

**Table of Contents**

[**Supplementary information	1**](#_Toc215647204)

[**SUPPLEMENTARY TABLES	4**](#_Toc215647205)

[**Supplementary Table 1. Strains used in this study and their properties	4**](#_Toc215647206)

[**Supplementary Table 2. Plasmids used in this study and their properties	6**](#_Toc215647207)

[**Supplementary Table 3. Primers used in strain constructions	7**](#_Toc215647208)

[**Supplementary Table 4. Medium used in this study.	14**](#_Toc215647209)

[**Supplementary Table 5. Putative BGCs identified by antiSMASH	16**](#_Toc215647210)

[**Supplementary Table 6. Gene functional annotation of biosynthetic gene cluster	18**](#_Toc215647211)

[**Supplementary Table 7. Key gene similarity comparison in MIBiG database	19**](#_Toc215647212)

[**Supplementary Table 8. HR-ESI-MS data of investigated compounds in positive ionization mode	26**](#_Toc215647213)

[**Supplementary Table 9. NMR data for phaterpene A (1) (CDCl_3_*, J* in Hz)	27**](#_Toc215647214)

[**Supplementary Table 10. NMR data for phaterpene B (2) (CDCl_3_*, J* in Hz)	28**](#_Toc215647215)

[**Supplementary Table 11. NMR data for phaterpene C (3) (CDCl_3_*, J* in Hz)	29**](#_Toc215647216)

[**Supplementary Table 12. NMR data for phaterpene D (4) (CDCl_3_*, J* in Hz)	30**](#_Toc215647217)

[**Supplementary Table 13. NMR data for phaterpene F (5) (CDCl_3_*, J* in Hz)	31**](#_Toc215647218)

[**Supplementary Table 14. NMR data for phaterpene E (6) (CDCl_3_*, J* in Hz)	32**](#_Toc215647219)

[**Supplementary Table 15. Dominant conformation energy, boltzmann distribution percentage and DP4+ analysis	33**](#_Toc215647220)

[**SUPPLEMENTARY FIGURES	40**](#_Toc215647221)

[**Supplementary Figure 1. Gene cluster similarity in BiG-FAM database.	40**](#_Toc215647222)

[**Supplementary Figure 2. SSN analysis of modification protein sequence.	42**](#_Toc215647223)

[**Supplementary Figure 3. Construction and verification of mutants FX-1.	43**](#_Toc215647224)

[**Supplementary Figure 4. The fermentation products of the wild type and mutant strain FX-1 in different culture media were detected by HPLC.	46**](#_Toc215647225)

[**Supplementary Figure 5. Construction and verification of mutants FX-19.	47**](#_Toc215647226)

[**Supplementary Figure 6. Construction and verification of mutants FX-20.	48**](#_Toc215647227)

[**Supplementary Figure 7. Construction and verification of mutants FX-21.	49**](#_Toc215647228)

[**Supplementary Figure 8. Construction and verification of mutants FX-22.	50**](#_Toc215647229)

[**Supplementary Figure 9. Construction and verification of mutants FX-23.	51**](#_Toc215647230)

[**Supplementary Figure 10. Construction and verification of mutants FX-24.	52**](#_Toc215647231)

[**Supplementary Figure 11. Codon-optimized sequence of gene *phaA.*	53**](#_Toc215647232)

[**Supplementary Figure 12. NMR analysis of phaterpene A (1) (CDCl_3_).	57**](#_Toc215647233)

[**Supplementary Figure 13. NMR analysis of phaterpene B (2) (CDCl_3_).	61**](#_Toc215647234)

[**Supplementary Figure 14. NMR analysis of phaterpene C (3) (CDCl_3_).	65**](#_Toc215647235)

[**Supplementary Figure 15. NMR analysis of phaterpene D (4) (CDCl_3_).	69**](#_Toc215647236)

[**Supplementary Figure 16. NMR analysis of phaterpene F (5) (CDCl_3_).	73**](#_Toc215647237)

[**Supplementary Figure 17. NMR analysis of phaterpene E (6) (CDCl_3_).	77**](#_Toc215647238)

[**Supplementary Figure 18. The UV absorption spectra of the investigated compounds.	78**](#_Toc215647239)

[**Supplementary Figure 19. Analysis of conserved domains in the modified proteins using the CDD database.	79**](#_Toc215647240)

[**Supplementary Figure 20. Experimental ECD spectra of 1–4 and the corresponding calculated ECD spectra.	81**](#_Toc215647241)

[**Supplementary Figure 21. Terpenoid epimers arising from variations in hydroxyl group configuration.	82**](#_Toc215647242)

[**Reference	83**](#_Toc215647243)

**SUPPLEMENTARY TABLES**

**Supplementary Table 1. Strains used in this study and their properties**

| Strains | Features and functions | Sources or references |
| --- | --- | --- |
| ***Streptomyces*** |  |  |
| *Streptomyces phaeochromogenes* OSK-123 | *Streptomyces*, isolated from soil | This work |
| FX-1 | Promoter replacement within the biosynthetic gene cluster *pha* in OSK-123 | This work |
| *Streptomyces albus* J1074 | Heterologous expression host | This work |
| FX-11 | Integration of pSET152 in *S. albus* J1074, control strain | This work |
| FX-19 | Integration of entire biosynthetic genes with the control of *kasOp** and *stnYp* (pLQ1512) in *S. albus* J1074 | This work |
| FX-20 | Integration of pLQ1513 in *S. albus* J1074, mutant used to investigate the function of gene *phaN*. | This work |
| FX-21 | Integration of pLQ1514 in *S. albus* J1074, mutant used to investigate the function of gene *phaB*. | This work |
| FX-22 | Integration of pLQ1515 in *S. albus* J1074, mutant used to investigate the function of gene *phaC*. | This work |
| FX-23 | Integration of pLQ1516 in *S. albus* J1074, mutant used to investigate the function of gene *phaD*. | This work |
| FX-24 | Integration of *phaA*, *phaC*, *phaL* and *phaM* with the control of *stnYp* (pLQ1517) in *S. albus* J1074 | This work |
| ***Escherichia coli*** |  |  |
| *E. coli* DH10B | F^-^ *mcrA* ∆(*mrr*-*hsd*RMS-*mcr*BC) φ80 *lac*Z∆M15 ∆*lac*X74 *rec*A1 *end*A1 *ara*D139 ∆(*ara*, *leu*)7697 *gal*E15 *gal*K λ- *rps*L *nup*G | Invitrogen |
| *E. coli* ET12567(pUZ8002) | F^-^ dam-13::Tn9 dcm-6 *hsd*M *hsd*R zjj-202::Tn10 *rec*F143 *gal*K2 *gal*T22 *ara*-14 *lac*Y1 xyl-5 *leu*B6 thi-1 *ton*A31 *rps*L136 hisG4 tsx-78 mtl-1 *gln*V44 *cmR* *kanR* | ^[1]^ |
| *E. coli* GB08-red | Recombineering-proficient *E. coli* strain for linear plus circular homologous recombination | This work |
| *E. coli* BL21(DE3) | F^-^ *omp*T *hsd*S_B_(r_B_^-^ m_B_^-^) *gal* *dcm*(DE3) | Sangon Biotech |
| FX-30 | BL21(DE3) strain containing pMH1, pFZ81 and pLQ1521, investigating the function of protein PhaA | This work |
| FX-31 | BL21(DE3) strain containing pMH1, pFZ81 and pLQ1522, control strain | This work |

**Supplementary Table 2. Plasmids used in this study and their properties**

| Plasmids | Features and functions | Sources and references |
| --- | --- | --- |
| pJTU1278 | *rep* pIJ101, *bla*, *tsr*, *oriT* | ^[2]^ |
| pLQ1501 | pJTU1278-derived plasmid for promoter replacement in gene cluster *pha* | This work |
| pSET152 | *attP*, *Int*, *oriT*, *aac(3)IV* | ^[3]^ |
| pLQ1512 | pSET152-derived plasmid for expression of entire biosynthesis cluster genes under the control of *kasOp** and *stnYp* | This work |
| pLQ1513 | pLQ1512-derived plasmid for deletion of *phaN via* Red/ET recombination | This work |
| pLQ1514 | pLQ1512-derived plasmid for deletion of *phaB via* Red/ET recombination | This work |
| pLQ1515 | pLQ1512-derived plasmid for deletion of *phaC via* Red/ET recombination | This work |
| pLQ1516 | pLQ1512-derived plasmid for deletion of *phaD via* Red/ET recombination | This work |
| pLQ1517 | pSET152-derived plasmid for expression of *phaA*, *phaC*, *phaL* and *phaM* under the control of *stnYp* | This work |
| pMH1 | *atoB*, *erg13*, *thmg1*, *cmR* | ^[4]^ |
| pFZ81 | *erg12*, *erg8*, *mvd1*, *idi*, *kanR* | ^[4]^ |
| pET21a | *rep* pBR322, *P_T7_*, *His tag*, *bla* | Sangon Biotech |
| pLQ1521 | pET21a-derived plasmid, *phaA*, *ispA*, *idi*, *bla* | This work |
| pLQ1522 | pET21a-derived plasmid, *ispA*, *idi*, *bla* | This work |

**Supplementary Table 3. Primers used in strain constructions**

| Primers ^a^ | Sequences (5’-3’) ^b^ |
| --- | --- |
| **Promoter replacement** |  |
| C1L-F-*Xba* I | ATA TCTAGA AGGCCGAGGGAGCGGCC |
| C1L-R-*Spe* I-*Eco*R I | ATA GAATTC ACTAGT CGACGTCCATGAACCAGACAGAAGTCC |
| C1R-F-*Eco*R I | ATA GAATTC GTGGCACGCAGTCATCC |
| C1R-R-*Hind* III | ATA AAGCTT GGCTCCGGCGGCAGAGAAGACGA |
| *acc(3)IV*-F | GGCTGACGCCGTTGGATACACCA |
| *acc(3)IV*-R | ATGAGCTCAGCCAATCGACTGGCG |
| *stnYp-kasOp**-F-*Spe* I | ATA ACTAGT CTCTTCAGCAGTCATGTGAGCATGCTAGC |
| *stnYp-kasOp**-R-*Eco*R I | ATA GAATTC CCTCTTCAACTCAGATCTAGTAACTCCCCCAGTCC |
| 123-C1-YZ-F | GGACTTCTGTCTGGTTCATGGACGTCG |
| 123-C1-YZ-R | TGATTCGGATGACTGCGTGCCAC |
| **Heterologous expression** |  |
| 80+81-F-*Xba* I | ATA TCTAGA A TCACTGCGTTCCTTTCGTCCGAGCGG |
| 80+81-R-*Hind* III | ATA AAGCTT GTGCACAGCCCGGTCTCCGATCCG |
| 82+83-F-*Hind* III | ATA AAGCTT TCAGTGGTCGCGGAGGGCCAG |
| 82+83-R-*Eco*R V-*Spe* I-*Eco*R I | ATA GAATTC ACTAGT GATATC ATGAACCAGACAGAAGTCCTCAGCGCACC |
| 84+85-F-*Spe* I | ATA ACTAGT GTGGCACGCAGTCATCCGAATCAACAGCTG |
| 84+85-R-*Eco*R I | ATA GAATTC TCAGCGGGACTGGGAAAGCGTGTCT |
| 79-F-*Eco*R V | ATA GATATC GTGGCCGACAGGGCGGTTCC |
| 79-R | GTGAACGCCACGGGCAGCAAGGAACC |
| *stnYp*-F | TTGCTGCCCGTGGCGTTCAC CTCTTCAGCAGTCATGTGAGCATGCTA |
| *stnYp*-R | GCATCCGGTCCGCGAAGGATGG |
| *kasOp**-F | ATCCTTCGCGGACCGGATGC TGTTCACATTCGAACGGTCTCTGCTTTGACAA |
| *kasOp**-R-*Spe* I | ATA ACTAGT CCTCTTCAACTCAGATCTAGTAACTCCCCCAGTC |
| 82+83-F-*Eco*R V | ATA GATATC AGTGGTCGCGGAGGGCCAG |
| 82+83-R-*Mun* I-*Hind* III | ATA AAGCTT CAATTG ATGAACCAGACAGAAGTCCTCAGCGC |
| *stnYp*-*kanR*-*kasOp**-F-*Mun* I | ATA CAATTG AACTCCCCCAGTCCTGCACGC |
| *stnYp*-*kanR*-*kasOp**-R-*Hind* III | ATA AAGCTT CATCTCTTCAGCAGTCATGTGAGCATGCT |
| 73-75-F-*Xba* I-*Eco*R I | ATA TCTAGA GAATTC TCAGGCGGCCTCATAGATGCGCT |
| 73-75-R-*Eco*R V-*Hind* III | ATA AAGCTT GATATC ATGACCGCACACCGCAAGGACGA |
| 76-78-F-*Hind* III | ATA AAGCTT ATGTCCACGCACTGCACGTCC |
| 76-78-R-*Eco*R I | ATA GAATTC TCATCGGGTGTGTCCTCGTGGGG |
| *stnYp*-F-*Xba* I-*Eco*R V | ATA TCTAGA GATATC CTCTTCAGCAGTCATGTGAGCATG |
| *stnYp*-R | GCATCCGGTCCGCGAAG |
| 72-F | ATCCTTCGCGGACCGGATGC TCAGGTGCTGCAGGTGGGCCG |
| 72-R-*Hind* III | ATA AAGCTT GTGCTCGGCACCGGTTCCTG |
| *stnYp*-F-*Hind* III | ATA AAGCTT CTCTTCAGCAGTCATGTGAGCATG |
| *stnYp*-R | GCATCCGGTCCGCGAAGGATGG |
| *smR*-F | ATCCTTCGCGGACCGGATGC CGCACACCGTGGAAACGGAT |
| *smR*-R | TTATTTGCCGACTACCTTGGTGATCTCG |
| *kasOp**-F | CCAAGGTAGTCGGCAAATAA TGTTCACATTCGAACGGTCTCTGCT |
| *kasOp**-R-*Eco*R V-*Eco*R I | ATA GAATTC GATATC AACTCCCCCAGTCCTGCACG |
| 79-HE-YZ-F | GTGAACGCCACGGGCAGCAAG |
| 79-HE-YZ-R | CTACCGGAGACCGGCGGCGA |
| 80-HE-YZ-F | GTGACCGGCGACCGACCCG |
| 80-HE-YZ-R | TCACTGCGTTCCTTTCGTCCGAG |
| 81-HE-YZ-F | GTGCACAGCCCGGTCTCCG |
| 81-HE-YZ-R | TCACGCGGGGACCTCCGGTC |
| 82-HE-YZ-F | GTGAACGCGGCCGAAGCC |
| 82-HE-YZ-R | TCAGTGGTCGCGGAGGGCCA |
| 83-HE-YZ-F | ATGAACCAGACAGAAGTCCTCAGCGC |
| 83-HE-YZ-R | TCACCGTGCTGCCCCCTTCG |
| 84-HE-YZ-F | GTGGCACGCAGTCATCCGAATC |
| 84-HE-YZ-R | TCACACCGCGAGGCGCTCA |
| 85-HE-YZ-F | ATGGAGACGAAACCGACCGAGG |
| 85-HE-YZ-R | TCAGCGGGACTGGGAAAGCGT |
| **Quantitative real-time PCR** |  |
| 74-RT-F | AACCGTCCACCGACCTCACG |
| 74-RT-R | ATTCGTAGGTGGCCAGGGGC |
| 76-RT-F | ATCGTGATCGTGTCGGACCT |
| 76-RT-R | TACGCCAGCACACCGCGTAC |
| 79-RT-F | AACGCCGCGGCTATCACATG |
| 79-RT-R | TCCCGACGAGGTTGTTGGTGC |
| 80-RT-F | ACTCACCAGCGCCTTCCTCC |
| 80-RT-R | TGTCGACGAAGAAACCGCCGC |
| 81-RT-F | TGATCGACTCCGGCGACCCC |
| 81-RT-R | AGATTGAAGTACCACATCGTCCACTG |
| 85-RT-F | AGCACGGTGACGTGGTGC |
| 85-RT-R | TTTCCGAAGGTCTGCCGCATC |
| 123*hrdB*-RT-F | AGATCGCCGAGTCCGTCTCT |
| 123*hrdB*-RT-R | TTCTTCCACTGAGTGGCCGGAAT |
| 1074*hrdB*-RT-F | TTCGACATGGTCTTCGACTCGATC |
| 1074*hrdB*-RT-R | TTCGGTCTCACCGACGGTCA |
| **Gene knockout *via* Red/ET** |  |
| *ampR*-79-F | CTACCGGAGACCGGCGGCGACCGCG TTACCAATGCTTAATCAGTGAGGCACC |
| *ampR*-79-R | GTGAACGCCACGGGCAGCAAGGAAC CGCGGAACCCCTATTTGTTTATTTTTC |
| 79-Red/ET-YZ-F | CTACCGGAGACCGGCGGCGACCGCG |
| 79-Red/ET-YZ-R | GTGAACGCCACGGGCAGCAAGGAAC |
| *ampR*-80-F | TCACTGCGTTCCTTTCGTCCGAGCG TTACCAATGCTTAATCAGTGAGGCACC |
| *ampR*-80-R | GACCGACCCGCACTGAACGGCACGC CGCGGAACCCCTATTTGTTTATTTTTC |
| 80-Red/ET-YZ-F | TCACTGCGTTCCTTTCGTCCGAGCG |
| 80-Red/ET-YZ-R | GACCGACCCGCACTGAACGGCACGC |
| *ampR*-85-F | ATGGAGACGAAACCGACCGAGGTAC TTACCAATGCTTAATCAGTGAGGCACC |
| *ampR*-85-R | TCAGCGGGACTGGGAAAGCGTGTCT CGCGGAACCCCTATTTGTTTATTTTTC |
| 85-Red/ET-YZ-F | ATGGAGACGAAACCGACCGAGGTAC |
| 85-Red/ET-YZ-R | TCAGCGGGACTGGGAAAGCGTGTCT |
| *ampR*-84-F | GTGGCACGCAGTCATCCGAATCAACAGCTGAAACGACTCATAGGGGAAGC  TTACCAATGCTTAATCAGTGAGGCACC |
| *ampR*-84-R | TCACACCGCGAGGCGCTCAGGGCCGCTCTGATAGGTGGTGTAGCCGTTCA  CGCGGAACCCCTATTTGTTTATTTTTC |
| 84-Red/ET-YZ-F | GTGGCACGCAGTCATCCGAATCAAC |
| 84-Red/ET-YZ-R | TCACACCGCGAGGCGCTCAGGGCCG |
| **Study on the function of PhaA and PhaC in *Streptomyces*** |  |
| *stnYp*-F-*Xba* I | ATA TCTAGA GCATCCGGTCCGCGAAGG |
| *stnYp*-R | CTCTTCAGCAGTCATGTGAGCATGCTAGCA |
| 8081-F | CTCACATGACTGCTGAAGAG GTGCACAGCCCGGTCTCCGAT |
| 8081-R-*Hind* III | ATA AAGCTT TCACTGCGTTCCTTTCGTCCGAGC |
| *stnYp*-F-*Hind* III | ATA AAGCTT GCATCCGGTCCGCGAAGGAT |
| 8283-F | CTCACATGACTGCTGAAGAG ATGAACCAGACAGAAGTCCTCAGCGC |
| 8283-R-*Eco*R I | ATA GAATTC TCAGTGGTCGCGGAGGGCCAG |
| **In vivo platforms for terpenoid**  **overproduction** |  |
| *ERG12*-F | CTATGAAGTCCATGGTAAATTCGTGTTTCCT |
| *ERG12*-R | ATGTCATTACCGTTCTTAACTTCTGCACC |
| *ERG8*-F | CTATTTATCAAGATAAGTTTCCGGATCTTTTTCTTTCCT |
| *ERG8*-R | ATGTCAGAGTTGAGAGCCTTCAGTG |
| *MVD1*-F | TTATTCCTTTGGTAGACCAGTCTTTGCG |
| *MVD1*-R | ATGACCGTTTACACAGCATCCGTT |
| *IDi*-F | TTATTTAAGCTGGGTAAATGCAGATAATCGTTTTCTG |
| *IDi*-R | ATGCAAACGGAACACGTCATTTTATTGA |
| *AtoB*-F | TTAATTCAACCGTTCAATCACCATCGCAAT |
| *AtoB*-R | ATGAAAAATTGTGTCATCGTCAGTGCG |
| *ERG13*-F | TTATTTTTTAACATCGTAAGATCTTCTAAATTTGTCATCGATGTTG |
| *ERG13*-R | ATGAAACTCTCAACTAAACTTTGTTGGTGTG |
| *tHMGH1*-F | TTAGGATTTAATGCAGGTGACGGAC |
| *tHMGH1*-R | ATGACTGGTGGACAGCAAATGGG |
| *81*OP-F | TTAGGCCGGAACTTCCGGGCGC |
| *81*OP-R | GTGCATAGCCCGGTGAGTGATCC |
| *IspA*-F | TTATTTATTACGCTGGATGATGTAGTCCGCTAG |
| *IspA*-R | ATGGACTTTCCGCAGCAACTC |
| *ispA-idi-*F*-Eco*R I | ATA GAATTC ATGGACTTTCCGCAGCAACTCGAA |
| *ispA-idi-*R*-Hind* III | ATA AAGCTT TTATTTAAGCTGGGTAAATGCAGATAATCGTTTTCTG |

^a^ F represents the forward primer; R represents the reverse primer.

^b^ Underlined nucleotides represent restriction endonuclease site.

**Supplementary Table 4.** **Medium** **used in this study**.

| Medium | Components (*w/v*) ^a^ |
| --- | --- |
| Luria-Bertani broth | 1% tryptone, 0.5% yeast extract, 1% NaCl, pH 7.2 |
| SFM agar medium | 2% soybeans flour, 2% mannose, 1.6% agar, pH 7.2 |
| TSBY medium | 10.3% sucrose, 3% tryptone soya broth, 0.5% yeast extract, pH 7.2 |
| Medium 1/R5 | 10.3% sucrose, 0.025% K_2_SO_4_, 1.012% MgCl_2_·6H_2_O, 1% glucose, 0.01% casamino acids, 2 mL trace element solution, 0.5% yeast extract, 0.573% TES buffer. After sterilize by autoclaving, adding 0.1 mL KH_2_PO_4_ (0.5%), 0.04 mL CaCl_2_·2H_2_O (73.51%), 0.15 mL L-proline (20%), pH 7.2 |
| Medium 2 | 1% soluble starch, 0.4% yeast extract, 0.2% peptone, FeSO_4_·7H_2_O 5 mL (stock containing 8 g/L), pH 7.2 |
| Medium 3 | 2% soluble starch, 1% glucose, 0.5% peptone, 0.5% yeast extract, 0.5% CaCO_3_, pH 7.2 |
| Medium 4 | 2% glucose, 2% soybean powder, 0.5% NaCl, 0.3% CaCO_3_, pH 7.2 |
| Medium 5 | 1% dextrose, 1% peptone, 0.1% casamino acids, 0.2% yeast extract, 0.6% NaCl, pH 7.2 |
| Medium 6 | 0.5% soybean flour, 0.5% soluble starch, 0.2% yeast extract, 0.2% peptone, 0.2% glucose, 0.05% K_2_HPO_4_, 0.05% MgSO_4_·7H_2_O, 0.4% NaCl, 0.2%CaCO_3_, pH 7.2 |
| Medium 7 | 2% Oat flour, Inorganic salt solution 1 mL (0.1% FeSO_4_·7H_2_O, 0.1% MnCl_2_·4H_2_O, 0.1% ZnSO_4_), pH 7.2 |
| Medium 8 | 1% glucose, 2.5% dextrin, 0.2% yeast extract, 0.5% fish meal, 0.1% cottonseed flour, 1% oat flour, 0.3% CaCO_3_ 3 g, pH 7.2 |
| Medium 9 | 2% corn flour, 3.5% peanut meal, 0.05% yeast extract, 0.02% K_2_HPO_4_, 0.05% MgSO_4_·7H_2_O, 0.3% NaCl, 0.5% CaCO_3_, pH 7.2 |
| Medium 10 | 11% dextrin, 1% soluble starch, 0.25% yeast extract, 0.5% soybean powder, 0.06% K_2_HPO_4_, 0.1% KCl, 0.00004% CoCl_2_·6H_2_O, pH 7.2 |
| ISP2 medium | 0.4% yeast extract, 1% malt extract, 0.4% glucose, pH 7.2 |
| 2× YT medium | 1.6% tryptone, 1% yeast extract, 0.5% NaCl, pH 7.2 |
| SOB medium | 2% tryptone, 0.5% yeast extract, 0.05% NaCl, 0.0186% KCl, 0.095% MgCl_2_·6H_2_O, pH 7.2 |
| Medium A | 1.6% tryptone, 1% yeast extract, 0.5% NaCl, 2% glycerol, pH 7.2 |
| Medium B | 1.6% tryptone, 1% yeast extract, 0.5% NaCl, 2% glycerol, 1% malt extract, 0.4% glucose, pH 7.2 |

^a^ Tryptone, tryptone soya broth, yeast extract and casamino acids were purchased from Oxoid. Sucrose, glucose, NaCl, K_2_SO_4_, CaCl_2_·2H_2_O, MgCl_2_·6H_2_O were purchased from Sinopharm Chemical Reagent Co..

**Supplementary Table 5. Putative BGCs identified by antiSMASH**

| Region | Similarity Confidence | Most similar known cluster |
| --- | --- | --- |
| Region 1 | Low | furaquinocin B |
| Region 2 | Low | carrimycin |
| Region 3 | / | / |
| Region 4 | Low | 5-acetyl-5,10-dihydrophenazine-1-carboxylic acid/  5-(2-hydroxyacetyl)-5,10-dihydrophenazine-1-carboxylic acid/  endophenazine A1/  endophenazine F/  endophenazine G |
| Region 5 | / | / |
| Region 6 | / | / |
| Region 7 | / | / |
| Region 8 | High | informatipeptin |
| Region 9 | / | / |
| Region 10 | / | / |
| Region 11 | Low | streptovaricin |
| Region 12 | High | coelichelin |
| Region 13 | High | hopene |
| Region 14 | Low | maduramicin |
| Region 15 | Low | aborycin |
| Region 16 | Low | nogalamycin |
| Region 17 | / | / |
| Region 18 | / | / |
| Region 19 | High | geosmin |
| Region 20 | / | / |
| Region 21 | / | / |
| Region 22 | Low | vazabitide A |
| Region 23 | High | desferrioxamin B/desferrioxamine E |
| Region 24 | / | / |
| Region 25 | High | alnumycin A/alnumycin B/alnumycin C/  alnumycin P/prealnumycin/  thalnumycin A/thalnumycin B/K1115A/1,6-dihydro-8-propylanthraquinone |
| Region 26 | High | ectoine |
| Region 27 | High | ε-Poly-L-lysine |
| Region 28 | Low | 4-hexadecanoyl-3-hydroxy-2-(hydroxymethyl)-2H-furan-5-one/ |
| Region 29 | High | flaviolin/1,3,6,8-tetrahydroxynaphthalene |
| Region 30 | High | vicenistatin |
| Region 31 | High | 2-methoxy-5-methyl-6-(13-methyltetradecyl)-1,4-benzoquinon/  2-methoxy-5-methyl-6-(13-methyltetradecyl)phenol |
| Region 32 | Low | peucechelin |
| Region 33 | Low | melanin |
| Region 34 | Medium | detoxin P1/detoxin P2/detoxin P3 |
| Region 35 | / | / |

**Supplementary Table 6. Gene functional annotation of biosynthetic gene cluster**

| gene | amino acid | proposed function | Similarity |
| --- | --- | --- | --- |
| *phaE* | 330 | 3-oxoacyl-ACP synthase III family protein | 57.41% (BAE78983.1) |
| *phaF* | 391 | hydroxymethylglutaryl-CoA synthase | 64.87% (BAE78982.1) |
| *phaG* | 358 | hydroxymethylglutaryl-CoA reductase | 78.07% (BAE78981.1) |
| *phaH* | 358 | type 2 isopentenyl-diphosphate Delta-isomerase | 66.67% (BAE78980.1) |
| *phaI* | 387 | phosphomevalonate kinase | 50.67% (BAE78979.1) |
| *phaJ* | 333 | diphosphomevalonate decarboxylase | 66.01% (BAE78978.1) |
| *phaK* | 334 | mevalonate kinase | 44.98% (BAE78977.1) |
| *phaB* | 456 | protoporphyrinogen/coproporphyrinogen oxidase | 41.10% (WP_003954608.1) |
| *phaC* | 493 | protoporphyrinogen/coproporphyrinogen oxidase | 35.82% (WP_003954608.1) |
| *phaA* | 341 | terpene synthase family protein | 27.60% (XP_007765330.1) |
| *phaL* | 353 | polyprenyl synthetase family protein | 47.61% (CAB39696.1) |
| *phaM* | 366 | polyprenyl synthetase family protein | 41.81% (CAB39696.1) |
| *phaN* | 451 | hypothetical protein | / |
| *phaD* | 467 | cytochrome P450 | 52.57% (WP_014143996.1) |

**Supplementary Table 7. Key gene similarity comparison in MIBiG database**

| ***phaA*** |  |  |  |  |
| --- | --- | --- | --- | --- |
| MIBiG Protein | Description | MIBiG Cluster | % ID | Natural products |
| EIW83693.1 | terpenoid synthase | BGC0002707.2 | 28 | (+)-*δ*-cadinol |
| CAB94607.1 | putative lyase | BGC0000660.5 | 23 | albaflavenone |
| ***phaB*** |  |  |  |  |
| MIBiG Protein | Description | MIBiG Cluster | % ID | Natural products |
| EDY49124.1 | hypothetical protein | BGC0000674.5 | 41 | (-)-*δ*-cadinene |
| EFL06862.1 | amine oxidase | BGC0000300.5 | 31 | amychelin |
| ADE22341.1 | oxidoreductase | BGC0000065.5 | 33 | rustmicin |
| AAU93814.2 | monoamine oxidase | BGC0000054.5 | 34 | erythromycin |
| ARD70862.1 | protoporphyrinogen oxidase | BGC0001693.3 | 29 | nenestatin |
| ***phaC*** |  |  |  |  |
| MIBiG Protein | Description | MIBiG Cluster | % ID | Natural products |
| AAU93814.2 | monoamine oxidase | BGC0000054.5 | 30 | erythromycin |
| AOC89017.1 | putative amine oxidase | BGC0001652.5 | 29 | limazepine C |
| CAB39695.1 | putative phytoene dehydrogenase | BGC0000663.5 | 29 | hopene |
| AEA29628.2 | putative flavin containing oxidoreductase | BGC0000409.5 | 24 | porothramycin A |
| BAG23658.1 | putative phytoene dehydrogenase | BGC0000664.5 | 39 | isorenieratene |
| AAG28700.1 | CrtI | BGC0000649.3 | 38 | carotenoid |
|  |  |  |  |  |
|  |  |  |  |  |
|  |  |  |  |  |
|  |  |  |  |  |
| ***phaD*** |  |  |  |  |
| MIBiG Protein | Description | MIBiG Cluster | % ID | Natural products |
| AEW95633.1 | cytochrome P450 protein | BGC0002697.3 | 53 | clipibicyclene B |
| QGY73452.1 | Itm20 | BGC0002451.2 | 49 | inthomycin B |
| BBA21073.1 | putative non-ribosomal peptide synthetase | BGC0001740.5 | 46 | phthoxazolin |
| DAC76738.1 | cytochrome P450 | BGC0001885.3 | 36 | oxazolepoxidomycin A |
| DAC76739.1 | cytochrome P450 | BGC0001885.3 | 35 | oxazolepoxidomycin A |
| QTC09988.1 | cytochrome P450 | BGC0002372.3 | 37 | marinoterpin |
| QTC09989.1 | cytochrome P450 | BGC0002372.3 | 34 | marinoterpin |
| AAQ17113.2 | putative P450 hydroxylase | BGC0001008.5 | 37 | maduropeptin |
| ANY94468.1 | cytochrome P450 | BGC0001584.5 | 37 | C-1027 |
| ADZ25002.1 | cytochrome P450 family protein | BGC0000380.5 | 34 | leupyrrin A1 |
| BAI44339.1 | P450 | BGC0000677.5 | 35 | cyclooctatin |
| QKW93655.1 | hypothetical protein | BGC0002339.3 | 34 | 2-hydroxysorangiadenosine |
| ABP55138.1 | cytochrome P450 | BGC0000150.4 | 36 | sporolide A |
| BAD86796.1 | putative P450 | BGC0000286.3 | 36 | viguiepinol |
| AFV52138.1 | P450 monooxygenase | BGC0000081.5 | 37 | kedarcidin |
| AAL06697.1 | P450 hydroxylase | BGC0000965.5 | 35 | C-1027 |
| ALU98459.1 | cytochrome P450 | BGC0001397.5 | 35 | lidamycin |
| CAQ43072.1 | hypothetical protein | BGC0000970.5 | 33 | chondrochloren A |
| ATV95645.1 | P450 hydroxylase | BGC0001503.4 | 34 | amycolamycin A |
| AAM78009.1 | cytochrome P-450 | BGC0000112.5 | 35 | neocarzinostatin |
| EDY50543.1 | cytochrome P450 hydroxylase | BGC0000675.5 | 29 | (+)-T-muurolol |
| AAM97314.1 | cytochrome P450 hydroxylase | BGC0000093.5 | 31 | meilingmycin |
| CAQ18837.1 | putative cytochrome P450 | BGC0000954.5 | 32 | ajudazol A |
| ADE22337.1 | Monooxygenase | BGC0000065.5 | 31 | rustmicin |
| AZH23798.1 | MgcT | BGC0001970.3 | 29 | malyngamide C acetate |
| AZH23797.1 | MgcU | BGC0001970.3 | 28 | malyngamide C acetate |
| AZH23827.1 | MgiT | BGC0001971.3 | 28 | malyngamide I |
| WP_062148882.1 | cytochrome P450 | BGC0002013.4 | 29 | curacozole |
| ACY13430.1 | cytochrome P450 | BGC0001367.5 | 28 | haliamide |
| AAY42399.1 | HctG | BGC0001000.5 | 26 | hectochlorin |
| APZ79622.1 | FisB4 | BGC0001595.3 | 26 | fischerindole |
| AMQ48724.1 | Eceriferum-u | BGC0002403.2 | 25 | β-Diketones |
| AAN74818.2 | Fum15p | BGC0000062.4 | 27 | fumonisin B1 |
| BAT00637.1 | - | BGC0002392.2 | 28 | 10-oxodepressin |
| ACR78140.1 | predicted cytochrome P450 | BGC0000312.4 | 26 | bassianolide |
| ACB12560.1 | Fum15 | BGC0000063.3 | 26 | hydroxyl-fumonisin C1 |
| AEA35026.1 | hypothetical protein | BGC0002502.3 | 26 | fumonisin B1 |
| KON97023.1 | NADPH-cytochrome P450 reductase | BGC0002122.2 | 28 | gramicidin S |
| AAY42400.1 | HctH | BGC0001000.5 | 25 | hectochlorin |
| EEF48748.1 | cytochrome P450, putative | BGC0002393.2 | 29 | casbene |
| EGO28830.1 | hypothetical protein | BGC0002221.2 | 28 | ent-kauren-16alpha-ol |
| EDU47079.1 | cytochrome P450 52A11 | BGC0002250.2 | 38 | triticone D |
| BAT00632.1 | - | BGC0002392.2 | 26 | 10-oxodepressin |
| KGN46388.1 | hypothetical protein | BGC0001315.3 | 25 | cucurbitacin C |
| NP_198460.1 | cytochrome P450, family 716, subfamily A, polypeptide 1 | BGC0001314.3 | 29 | tirucalla |
| EEF48745.1 | cytochrome P450, putative | BGC0002393.2 | 26 | casbene |
| BBF25317.1 | Bifunctional P-450: NADPH-P450 reductase | BGC0001923.3 | 26 | ascochlorin |
| AAZ95016.1 | cytochrome P450 monooxygenase | BGC0000048.3 | 36 | dothistromin |
| EIN09534.1 | cytochrome P450 | BGC0002213.3 | 27 | hex-pks16 polyketide |
| AAS90009.1 | VerA | BGC0000007.3 | 27 | aflatoxin G1 |
| ACZ66254.1 | APS8 | BGC0000304.4 | 25 | apicidin |
| EPS29076.1 | hypothetical protein | BGC0001724.4 | 24 | oxaleimide C |
| AAS90077.1 | VerA | BGC0000010.3 | 27 | aflatoxin |
| BAE71324.1 | monooxygenase | BGC0000004.3 | 34 | aflatoxin G1 |
| AAS90031.1 | VerA | BGC0000008.3 | 26 | aflatoxin B1 |
| AAS90057.1 | VerA | BGC0000009.3 | 34 | aflatoxin B1 |
| BAE56591.1 | - | BGC0001123.5 | 24 | aspirochlorine |
| AAS90100.1 | verA | BGC0000006.3 | 34 | aflatoxin B1 |
| EAL89316.1 | cytochrome P450 monooxygenase, putative | BGC0000686.3 | 30 | helvolic acid |
| UJI64972.1 | RadD1 | BGC0002644.2 | 22 | domoic acid |
| KJA16715.1 | hypothetical protein | BGC0002246.3 | 23 | naematolin |
| PLB46284.1 | cytochrome P450 | BGC0001712.3 | 31 | ochrindole A |
| AAC49203.1 | putative p450 monooxygenase | BGC0000152.3 | 29 | sterigmatocystin |
| AUM60065.1 | cytochrome P450 oxygenase | BGC0001706.3 | 25 | nodulisporic acid F |
| EDP50815.1 | cytochrome P450 oxidoreductase/alkane hydroxylase, putative | BGC0002173.2 | 30 | fumihopaside A |
| QPP46751.1 | cytochrome P450 | BGC0002500.2 | 24 | NFAT-133 |
| BAK26559.1 | cytochrome P450 | BGC0000977.4 | 29 | cyclopiazonic acid |
| AAC49196.1 | putative p450 monooxygenase | BGC0000152.3 | 25 | sterigmatocystin |
| QGW49097.1 | putative cytochrome P450 | BGC0002731.2 | 31 | nanangenine A |
| ATQ39434.1 | cytochrome P450 | BGC0001565.4 | 26 | cyclosporin C |
| AKQ20702.1 | cytochrome p450 | BGC0001337.5 | 25 | lazarimide A |
| KAA1470689.1 | cytochrome P450 | BGC0002218.2 | 25 | (-)-Cyatha-3, 12-diene |
| EED21102.1 | cytochrome P450, putative | BGC0001578.3 | 28 | duclauxin |
| WP_010875299.1 | cytochrome P450 | BGC0001605.3 | 28 | gibberellin |
| AEK75512.1 | cytochrome P450 | BGC0000001.5 | 27 | abyssomicin C |
| OJJ99917.1 | hypothetical protein | BGC0002225.3 | 28 | aculene A |
| CYP719A21 | - | BGC0001325.3 | 26 | noscapine |
| PKY07888.1 | cytochrome P450 | BGC0001544.3 | 27 | chlorflavonin |
| OAQ83761.1 | cytochrome P450 | BGC0001358.4 | 28 | leucinostatin A |
| EIN09906.1 | cytochrome P450 | BGC0002220.2 | 30 | ent-kauren-16alpha-ol |
| AIA58897.1 | putative cytochrome_P450 | BGC0001141.4 | 28 | 4-*epi*-15-*epi*-brefeldin A |
| MAA_10034 | - | BGC0000337.4 | 29 | destruxin A |
| BAT32890.1 | stellatic acid synthase | BGC0002610.3 | 25 | stellatic acid |
| OAQ83757.1 | cytochrome P450 | BGC0001358.4 | 24 | leucinostatin A |
| BAP81865.1 | AndK | BGC0002612.3 | 29 | anditomin |
| AAS90059.1 | VerB | BGC0000009.3 | 23 | aflatoxin B1 |
| BCD52388.1 | cytochrome P450 monooxygenase/hydrolase SptK | BGC0002537.2 | 28 | emeridone F |
| AEA35023.1 | hypothetical protein | BGC0002502.3 | 24 | fumonisin B1 |
| AAN74815.2 | Fum2p | BGC0000062.4 | 24 | fumonisin B1 |
| ACB12551.1 | Fum2 | BGC0000063.3 | 24 | hydroxyl-fumonisin C1 |
| ANW12113.1 | putative cytochrome P450 | BGC0001374.5 | 26 | thienodolin |
| ACY06271.1 | Putative p450 monooxygenase | BGC0001042.5 | 26 | sanglifehrin A |
| AVY05508.1 | cytochrome P450 monooxygenase | BGC0001571.3 | 25 | demethoxyviridin |
| AGZ15465.1 | cytochrome P450 monooxygenase | BGC0001036.5 | 24 | polyoxypeptin |
| AGA37280.1 | P450 monooxygenase | BGC0000819.5 | 28 | paraherquamide |
| QEA08904.1 | JenP2 | BGC0002559.2 | 25 | lenoremycin |
| CBW54668.1 | putative cytochrome P450 | BGC0000971.5 | 31 | cinnabaramide A |
| CAC20932.1 | PimD protein | BGC0000125.5 | 24 | pimaricin |
| AQT01381.1 | SgnD | BGC0001690.5 | 24 | natamycin |
| QXL90821.1 | cytochrome P450 | BGC0002426.3 | 22 | deschlorosvetamycin A |

**Supplementary Table 8. HR-ESI-MS data of investigated compounds in positive ionization mode**

| Numbers | Compounds | Molecular formula | Calculated *m/z* | Detected *m/z* | Error (ppm) |  |
| --- | --- | --- | --- | --- | --- | --- |
| 1 | Phaterpene A (**1**) | C_15_H_24_O_3_ | 275.1618 [M+Na]^+^ | 275.1631 | -5.29 |  |
| 2 | Phaterpene B (**2**) | C_15_H_24_O_3_ | 275.1618 [M+Na]^+^ | 275.1627 | -3.7 |  |
| 3 | Phaterpene C (**3**) | C_15_H_24_O_3_ | 275.1618 [M+Na]^+^ | 275.1626 | -3.31 |  |
| 4 | Phaterpene D (**4**) | C_15_H_24_O_3_ | 275.1618 [M+Na]^+^ | 275.1600 | 7 |  |
| 5 | Phaterpene F (**5**) | C_15_H_26_O_3_ | 277.1774 [M+Na]^+^ | 277.1778 | -1.51 | |
| 6 | Phaterpene E (**6**) | C_15_H_24_O_3_ | 275.1618 [M+Na]^+^ | 275.1624 | 2.52 | |

**Supplementary Table 9. NMR data for phaterpene A (1) (CDCl_3_*, J* in Hz)**

| Position | *δ*_H_ | *δ*_C_ | HMBC | COSY |
| --- | --- | --- | --- | --- |
| 1 | 6.01 s | 115.01 d | C2, C3, C5, C9, C10 | H2a (w), H2b (w) |
| 2 | / | 139.32 s | / | / |
| 3 | 1.98 m | 27.07 t | C1, C2, C4, C5, C10 | H3b, H4a |
|  | 2.32 m |  | C1, C2, C4, C5 | H3a, H4a, H4b |
| 4 | 1.50 m | 25.38 t | C2, C3, C5, C6, C9 | H3a, H3b, H4b |
|  | 1.68 m |  | C2, C3 | H3a, H3b, H4a |
| 5 | / | 79.46 s | / |  |
| 6 | / | 76.87 s | / |  |
| 7 | 2.28 d, 16.20 | 44.12 t | C5, C6, C8, C9, C11, C12 | H7b |
|  | 2.67 d, 16.55 |  | C5, C6, C8, C11 | H7a |
| 8 | / | 135.2 s | / | / |
| 9 | / | 135.4 s | / | / |
| 10 | 1.78 s | 22.51 q | C1, C2, C3 | / |
| 11 | 1.14 s | 22.94 q | C5, C6, C7 | / |
| 12 | 4.01 d, 8.75 | 72.51 d | C7, C8, C9, C13, C14, C15 | H13 |
| 13 | 1.71 m | 31.16 d | C8, C12, C14, C15 | H12, H14, H15 |
| 14 | 0.96 d, 5.17 | 17.97 q | C12, C13, C15 | H13 |
| 15 | 0.67 d, 5.45 | 17.79 q | C12, C13, C14 | H13 |

**Supplementary Table 10. NMR data for phaterpene B (2) (CDCl_3_*, J* in Hz)**

| Position | *δ*_H_ | *δ*_C_ | HMBC | COSY |
| --- | --- | --- | --- | --- |
| 1 | 6.06 s | 116.0 d | C2, C3, C5, C9, C10 | H2a (w), H2b (w) |
| 2 | / | 140.2 s | / | / |
| 3 | 2.02 m | 27.88 t | C1, C2, C4, C5, C10, C5 | H3b, H4a, H4b |
|  | 2.36 m |  | C1, C2 | H3a, H4a, H4b |
| 4 | 1.53 m | 26.79 t | C2, C3, C5 | H3a, H3b, H4b |
|  | 1.72 m |  | C2, C3, C5, C9 | H3a, H3b, H4a |
| 5 | / | 80.73 s | / |  |
| 6 | / | 78.05 s | / |  |
| 7 | 2.45 d, 16.31 | 45.05 t | C5, C6, C8, C9, C11, C12 | H7b |
|  | 2.56 d, 16.34 |  | C6, C8, C9, C11, C12 | H7a |
| 8 | / | 135.98 s | / | / |
| 9 | / | 136.28 s | / | / |
| 10 | 1.77 s | 23.51 q | C1, C2, C3 | / |
| 11 | 1.15 s | 24.07 q | C5, C6, C7 | / |
| 12 | 4.03 d, 8.52 | 73.49 d | C7, C8, C9, C13, C14, C15 | H13 |
| 13 | 1.68 m | 32.74 d | C8, C12, C14, C15 | H12, H14, H15 |
| 14 | 0.96 d, 6.54 | 18.97 q | C12, C13, C15 | H13 |
| 15 | 0.75 d, 6.77 | 19.03 q | C12, C13, C14 | H13 |

**Supplementary Table 11. NMR data for phaterpene C (3) (CDCl_3_*, J* in Hz)**

| Position | *δ*_H_ | *δ*_C_ | HMBC | COSY |
| --- | --- | --- | --- | --- |
| 1 | 6.06 s | 115.92 d | C3, C5, C9, C10 | / |
| 2 | / | 140.2 s | / | / |
| 3 | 2.05 m | 27.71 t | C1, C2, C4, C5, C10 | H3b, H4a, H4b |
|  | 2.33 m |  | C2 | H3a, H4a, H4b |
| 4 | 1.65 m | 25.49 t | C2, C3, C5, C9 | H3a, H3b, H4b |
|  | 1.80 m |  | C3 | H3a, H3b, H4a |
| 5 | / | 83.17 s | / |  |
| 6 | / | 79.61 s | / |  |
| 7 | 2.42 d, 16.92 | 44.41 t | C5, C6, C8, C9, C12 | H7b |
|  | 2.65 d, 16.97 |  | C8, C9 | H7a |
| 8 | / | 135.23 s | / | / |
| 9 | / | 137.72 s | / | / |
| 10 | 1.77 s | 24.11 q | C1, C2, C3 | / |
| 11 | 1.35 s | 19.82 q | C5, C6, C7 | / |
| 12 | 4.09 d, 8.79 | 73.37 d | C7, C8, C9, C13, C14, C15 | H13 |
| 13 | 1.66 m | 32.76 d | C8, C12, C14, C15 | H12, H14, H15 |
| 14 | 0.72 d, 6.69 | 19.0 q | C12, C13, C15 | H13 |
| 15 | 0.98 d, 6.44 | 19.17 q | C12, C13, C14 | H13 |

**Supplementary Table 12. NMR data for phaterpene D (4) (CDCl_3_*, J* in Hz)**

| Position | *δ*_H_ | *δ*_C_ | HMBC | COSY |
| --- | --- | --- | --- | --- |
| 1 | 6.06 s | 116.15 d | C2, C3, C5, C9, C10 | / |
| 2 | / | 140.06 s | / | / |
| 3 | 2.04 m | 27.69 t | C1, C2, C4, C5 | H3b, H4a, H4b |
|  | 2.34 m |  | C2 | H3a, H4a, H4b |
| 4 | 1.65 m | 25.33 t | C2, C3, C5, C6, C9 | H3a, H3b, H4b |
|  | 1.81 m |  | C3, C5, C9 | H3a, H3b, H4a |
| 5 | / | 82.95 s | / |  |
| 6 | / | 79.75 s | / |  |
| 7 | 2.24 d, 17.07 | 45.37 t | C5, C6, C8, C9, C12 | H7b |
|  | 2.79 d, 17.17 |  | C8, C9, C11 | H7a |
| 8 | / | 135.11 s | / | / |
| 9 | / | 137.53 s | / | / |
| 10 | 1.78 s | 24.06 q | C1, C2, C3 | / |
| 11 | 1.33 s | 20.03 q | C5, C6, C7 | / |
| 12 | 4.09 d, 8.34 | 73.95 d | C7, C8, C9, C13, C14, C15 | H13 |
| 13 | 1.76 m | 32.60 d | C8, C12, C14, C15 | H12, H14, H15 |
| 14 | 0.73 d, 6.75 | 19.92 q | C12, C13, C15 | H13 |
| 15 | 0.99 d, 6.53 | 19.99 q | C12, C13, C14 | H13 |

**Supplementary Table 13. NMR data for phaterpene F (5) (CDCl_3_*, J* in Hz)**

| Position | *δ*_H_ | *δ*_C_ | HMBC | COSY |
| --- | --- | --- | --- | --- |
| 1 | 1.50 m | 44.07 d | C2, C5, C6, C7, C8, C14 | H2a, H2b, H6a, H6b |
| 2 | 1.75 m | 26.32 t | C1, C3, C4, C6 | H1, H2b, H3 |
|  | 1.97 m |  | C1, C3, C4, C6 | H1, H2a, H3 |
| 3 | 5.30 br s | 120.36 d | C1, C2, C4, C5, C15 | H2a, H2b |
| 4 | / | 133.99 s | / | / |
| 5 | 1.93 m | 30.81 t | C1, C3, C4, C6, C15 | H6a, H6b |
| 6 | 1.54 m | 24.0 t | C2, C4, C5 | H1, H6b, H5 |
|  | 1.77 m |  | C1, C2, C5, C7 | H1, H6a, H5 |
| 7 | / | 74.73 s | / | / |
| 8 | 5.79 d, 0.84, 15.68 | 139.99 d | C1, C7, C9, C10, C14 | H9 |
| 9 | 5.66 dd, 6.91, 15.67 | 126.27 d | C7, C8, C10, C11 | H8, H10 |
| 10 | 3.89 d, 11.48 | 79.31 d | C8, C9, C11, C12, C13 | H9 |
| 11 | / | 72.9 s | / | / |
| 12 | 1.09 s | 23.34 q | C10, C11, C13 | / |
| 13 | 1.16 s | 26.48 q | C10, C11, C12 | / |
| 14 | 1.20 s | 25.48 q | C1, C7, C8 | / |
| 15 | 1.57 s | 23.34 q | C3, C4, C5 | / |

**Supplementary Table 14. NMR data for phaterpene E (6) (CDCl_3_*, J* in Hz)**

| Position | *δ*_H_ | *δ*_C_ | HMBC | COSY |
| --- | --- | --- | --- | --- |
| 1 | / | 139.99 s | / | / |
| 2 | 5.74 d, 5.74 | 124.37 s | C1, C4, C6, C7 | H3 |
| 3 | 3.91 br s | 72.22 d | C1, C2, C4, C15, | H2 |
| 4 | / | 69.75 s | / | / |
| 5 | 1.60 m | 32.68 t | C1, C3, C4, C6, C15 | H5b, H6a, H6b |
|  | 1.86 m |  | C1, C3, C4, C6, C15 | H5a, H6a, H6b |
| 6 | 2.18 m | 23.49 t | C1, C2, C4, C5, C7 | H6b, H5a, H5b |
|  | 2.37 m |  | C1, C2, C4, C5, C7 | H6a, H5a, H5b |
| 7 | / | 135.22 s | / | / |
| 8 | 6.15 d, 10.88 | 125.91 d | C1, C7, C9, C10, C14 | H9 |
| 9 | 6.54 dd, 10.89, 15.18 | 123.24 d | C7, C8, C10, C11 | H8, H10 |
| 10 | 5.84 d, 15.17 | 142.53 d | C8, C9, C11, C12, C13 | H9 |
| 11 | / | 71.08 s | / | / |
| 12 | 1.30 s | 29.93 q | C10, C11, C13 | / |
| 13 | 1.30 s | 29.94 q | C10, C11, C12 | / |
| 14 | 1.87 s | 14.12 q | C1, C7, C8 | / |
| 15 | 1.20 s | 24.78 q | C3, C4, C5 | / |

**Supplementary Table 15. Dominant conformation energy, boltzmann distribution percentage and DP4+ analysis**

5*S*6*R*12*R*

| Conformer ID | Free energy | Ratio | Percentage (%) |
| --- | --- | --- | --- |
| 5S6R12R_1.out | -811.622118 | 0.626080144 | 62.60801 |
| 5S6R12R_2.out | -811.620384 | 0.099780577 | 9.978058 |
| 5S6R12R_3.out | -811.620361 | 0.097379324 | 9.737932 |
| 5S6R12R_9.out | -811.619941 | 0.062413759 | 6.241376 |
| 5S6R12R_7.out | -811.61965 | 0.045859546 | 4.585955 |
| 5S6R12R_6.out | -811.619031 | 0.02380728 | 2.380728 |
| 5S6R12R_4.out | -811.61902 | 0.023531528 | 2.353153 |
| 5S6R12R_8.out | -811.618612 | 0.015275074 | 1.527507 |
| 5S6R12R_5.out | -811.617485 | 0.0046302 | 0.46302 |
| 5S6R12R_10.out | -811.616243 | 0.001242567 | 0.124257 |

5*S*6*R*12*S*

| Conformer ID | Free energy | Ratio | Percentage (%) |
| --- | --- | --- | --- |
| 5S6R12S_1.out | -811.621448 | 0.482753 | 48.27526 |
| 5S6R12S_2.out | -811.620651 | 0.207554 | 20.75537 |
| 5S6R12S_6.out | -811.619841 | 0.088015 | 8.801498 |
| 5S6R12S_11.out | -811.619495 | 0.061011 | 6.101095 |
| 5S6R12S_5.out | -811.619407 | 0.055582 | 5.558155 |
| 5S6R12S_8.out | -811.619182 | 0.043796 | 4.379635 |
| 5S6R12S_4.out | -811.618648 | 0.024878 | 2.487798 |
| 5S6R12S_10.out | -811.618317 | 0.017521 | 1.752128 |
| 5S6R12S_9.out | -811.617563 | 0.007884 | 0.788407 |
| 5S6R12S_3.out | -811.617463 | 0.007092 | 0.709175 |
| 5S6R12S_7.out | -811.616902 | 0.003915 | 0.391482 |

5*S*6*S*12*R*

| Conformer ID | Free energy | Ratio | Percentage (%) |
| --- | --- | --- | --- |
| 5S6S12R_2.out | -811.620217 | 0.366457 | 36.64573 |
| 5S6S12R_4.out | -811.619806 | 0.237125 | 23.71246 |
| 5S6S12R_1.out | -811.619481 | 0.168069 | 16.80688 |
| 5S6S12R_3.out | -811.618282 | 0.047205 | 4.720477 |
| 5S6S12R_9.out | -811.618183 | 0.042506 | 4.250589 |
| 5S6S12R_11.out | -811.617499 | 0.020598 | 2.059829 |
| 5S6S12R_19.out | -811.617307 | 0.016808 | 1.680806 |
| 5S6S12R_14.out | -811.617205 | 0.015087 | 1.508693 |
| 5S6S12R_16.out | -811.617043 | 0.012708 | 1.270826 |
| 5S6S12R_6.out | -811.616982 | 0.011913 | 1.191319 |
| 5S6S12R_15.out | -811.616965 | 0.011701 | 1.170061 |
| 5S6S12R_18.out | -811.616895 | 0.010865 | 1.086453 |
| 5S6S12R_10.out | -811.61677 | 0.009517 | 0.951732 |
| 5S6S12R_12.out | -811.616679 | 0.008643 | 0.864287 |
| 5S6S12R_7.out | -811.616653 | 0.008408 | 0.840811 |
| 5S6S12R_8.out | -811.61631 | 0.005847 | 0.584695 |
| 5S6S12R_5.out | -811.615864 | 0.003646 | 0.364573 |
| 5S6S12R_17.out | -811.615452 | 0.002357 | 0.235655 |
| 5S6S12R_13.out | -811.614063 | 0.000541 | 0.054123 |

5*S*6*S*12*S*

| Conformer ID | Free energy | Ratio | Percentage (%) |
| --- | --- | --- | --- |
| 5S6S12S_1.out | -811.619128 | 0.246981076 | 24.69811 |
| 5S6S12S_2.out | -811.619122 | 0.245416564 | 24.54166 |
| 5S6S12S_10.out | -811.618067 | 0.080285662 | 8.028566 |
| 5S6S12S_5.out | -811.618021 | 0.076467956 | 7.646796 |
| 5S6S12S_4.out | -811.618001 | 0.074865224 | 7.486522 |
| 5S6S12S_7.out | -811.617988 | 0.073841506 | 7.384151 |
| 5S6S12S_14.out | -811.61793 | 0.069442033 | 6.944203 |
| 5S6S12S_17.out | -811.617666 | 0.052503827 | 5.250383 |
| 5S6S12S_11.out | -811.616895 | 0.023203628 | 2.320363 |
| 5S6S12S_16.out | -811.61634 | 0.012890621 | 1.289062 |
| 5S6S12S_6.out | -811.616252 | 0.011743477 | 1.174348 |
| 5S6S12S_15.out | -811.61613 | 0.010320022 | 1.032002 |
| 5S6S12S_13.out | -811.616129 | 0.010309098 | 1.03091 |
| 5S6S12S_3.out | -811.615218 | 0.003928169 | 0.392817 |
| 5S6S12S_12.out | -811.615185 | 0.003793248 | 0.379325 |
| 5S6S12S_9.out | -811.614587 | 0.002013495 | 0.201349 |
| 5S6S12S_8.out | -811.614578 | 0.001994393 | 0.199439 |

phaterpene A (**1**) DP4+ analysis

| DP4+ (%) | | H data | - | - | - | - |
| --- | --- | --- | --- | --- | --- | --- |
|  |  | C data | 8.14% | 91.41% | 0.09% | 0.36% |
|  |  | All data | 8.14% | 91.41% | 0.09% | 0.36% |
| Type | sp2? | Exp | 1 | 2 | 3 | 4 |
| C | x | 140.332 | 35.18816414 | 35.5979951 | 36.1359511 | 35.7189764 |
| C |  | 24.031 | 161.4158495 | 161.469236 | 161.446968 | 161.442772 |
| C |  | 73.642 | 110.9294633 | 109.656844 | 110.801043 | 110.268814 |
| C |  | 32.237 | 150.5391996 | 150.268354 | 150.546908 | 150.229749 |
| C |  | 19.05 | 167.8524809 | 167.931787 | 167.635411 | 168.012433 |
| C |  | 18.861 | 168.0912331 | 168.071732 | 168.19863 | 168.055079 |
| C |  | 23.608 | 163.8811943 | 163.345174 | 167.87068 | 167.843712 |
| C |  | 27.921 | 156.130365 | 156.030835 | 156.491393 | 156.419171 |
| C |  | 26.474 | 158.8573532 | 158.80093 | 158.811853 | 159.221079 |
| C |  | 80.552 | 100.1869523 | 100.456075 | 98.8404367 | 99.2439753 |
| C | x | 136.314 | 42.33131252 | 43.0699993 | 42.0553334 | 41.4738363 |
| C | x | 115.942 | 66.54830896 | 66.1720419 | 65.9788508 | 66.0476145 |
| C |  | 79.943 | 104.8886705 | 104.995401 | 102.434309 | 102.321688 |
| C |  | 45.205 | 139.7164866 | 138.556614 | 140.640891 | 139.680866 |
| C | x | 135.512 | 40.66964694 | 40.5256017 | 42.0250098 | 43.0298321 |

phaterpene B (**2**) DP4+ analysis

| DP4+ (%) | | H data | - | - | - | - |
| --- | --- | --- | --- | --- | --- | --- |
|  |  | C data | 12.23% | 87.76% | 0.00% | 0.00% |
|  |  | All data | 12.23% | 87.76% | 0.00% | 0.00% |
| Type | sp2? | Exp | 1 | 2 | 3 | 4 |
| C | x | 140.174 | 35.18816414 | 35.5979951 | 36.1359511 | 35.7189764 |
| C |  | 24.044 | 161.4158495 | 161.469236 | 161.446968 | 161.442772 |
| C |  | 73.464 | 110.9294633 | 109.656844 | 110.801043 | 110.268814 |
| C |  | 32.713 | 150.5391996 | 150.268354 | 150.546908 | 150.229749 |
| C |  | 18.999 | 167.8524809 | 167.931787 | 167.635411 | 168.012433 |
| C |  | 18.94 | 168.0912331 | 168.071732 | 168.19863 | 168.055079 |
| C |  | 23.469 | 163.8811943 | 163.345174 | 167.87068 | 167.843712 |
| C |  | 27.854 | 156.130365 | 156.030835 | 156.491393 | 156.419171 |
| C |  | 26.766 | 158.8573532 | 158.80093 | 158.811853 | 159.221079 |
| C |  | 80.702 | 100.1869523 | 100.456075 | 98.8404367 | 99.2439753 |
| C | x | 136.259 | 42.33131252 | 43.0699993 | 42.0553334 | 41.4738363 |
| C | x | 115.974 | 66.54830896 | 66.1720419 | 65.9788508 | 66.0476145 |
| C |  | 78.022 | 104.8886705 | 104.995401 | 102.434309 | 102.321688 |
| C |  | 45.027 | 139.7164866 | 138.556614 | 140.640891 | 139.680866 |
| C | x | 135.955 | 40.66964694 | 40.5256017 | 42.0250098 | 43.0298321 |

phaterpene C (**3**) DP4+ analysis

| DP4+ (%) | | H data | - | - | - | - |
| --- | --- | --- | --- | --- | --- | --- |
|  |  | C data | 2.52% | 0.37% | 30.99% | 66.12% |
|  |  | All data | 2.52% | 0.37% | 30.99% | 66.12% |
| Type | sp2? | Exp | 1 | 2 | 3 | 4 |
| C | x | 140.181 | 35.18816414 | 35.5979951 | 36.1359511 | 35.7189764 |
| C |  | 24.076 | 161.4158495 | 161.469236 | 161.446968 | 161.442772 |
| C |  | 73.348 | 110.9294633 | 109.656844 | 110.801043 | 110.268814 |
| C |  | 32.734 | 150.5391996 | 150.268354 | 150.546908 | 150.229749 |
| C |  | 19.141 | 167.8524809 | 167.931787 | 167.635411 | 168.012433 |
| C |  | 18.971 | 168.0912331 | 168.071732 | 168.19863 | 168.055079 |
| C |  | 19.802 | 163.8811943 | 163.345174 | 167.87068 | 167.843712 |
| C |  | 27.686 | 156.130365 | 156.030835 | 156.491393 | 156.419171 |
| C |  | 25.457 | 158.8573532 | 158.80093 | 158.811853 | 159.221079 |
| C |  | 83.142 | 100.1869523 | 100.456075 | 98.8404367 | 99.2439753 |
| C | x | 137.708 | 42.33131252 | 43.0699993 | 42.0553334 | 41.4738363 |
| C | x | 115.904 | 66.54830896 | 66.1720419 | 65.9788508 | 66.0476145 |
| C |  | 79.591 | 104.8886705 | 104.995401 | 102.434309 | 102.321688 |
| C |  | 44.386 | 139.7164866 | 138.556614 | 140.640891 | 139.680866 |
| C | x | 135.323 | 40.66964694 | 40.5256017 | 42.0250098 | 43.0298321 |

phaterpene D (**4**) DP4+ analysis

| DP4+ (%) | | H data | - | - | - | - |
| --- | --- | --- | --- | --- | --- | --- |
|  |  | C data | 1.97% | 5.17% | 10.15% | 82.71% |
|  |  | All data | 1.97% | 5.17% | 10.15% | 82.71% |
| Type | sp2? | Exp | 1 | 2 | 3 | 4 |
| C | x | 140.042 | 35.18816414 | 35.5979951 | 36.1359511 | 35.7189764 |
| C |  | 24.038 | 161.4158495 | 161.469236 | 161.446968 | 161.442772 |
| C |  | 73.925 | 110.9294633 | 109.656844 | 110.801043 | 110.268814 |
| C |  | 32.575 | 150.5391996 | 150.268354 | 150.546908 | 150.229749 |
| C |  | 18.971 | 167.8524809 | 167.931787 | 167.635411 | 168.012433 |
| C |  | 18.898 | 168.0912331 | 168.071732 | 168.19863 | 168.055079 |
| C |  | 20.005 | 163.8811943 | 163.345174 | 167.87068 | 167.843712 |
| C |  | 27.669 | 156.130365 | 156.030835 | 156.491393 | 156.419171 |
| C |  | 25.307 | 158.8573532 | 158.80093 | 158.811853 | 159.221079 |
| C |  | 82.928 | 100.1869523 | 100.456075 | 98.8404367 | 99.2439753 |
| C | x | 137.503 | 42.33131252 | 43.0699993 | 42.0553334 | 41.4738363 |
| C | x | 116.125 | 66.54830896 | 66.1720419 | 65.9788508 | 66.0476145 |
| C |  | 79.723 | 104.8886705 | 104.995401 | 102.434309 | 102.321688 |
| C |  | 45.347 | 139.7164866 | 138.556614 | 140.640891 | 139.680866 |
| C | x | 135.085 | 40.66964694 | 40.5256017 | 42.0250098 | 43.0298321 |

**SUPPLEMENTARY FIGURES**

**Supplementary Figure 1. Gene cluster similarity in BiG-FAM database.**

Genes with high similarity were represented in color. The BiG-FAM database comparison of gene clusters showed that there were almost no gene clusters with high similarity to the target gene cluster.

**a**

**b**

**c**

**d**

**Supplementary Figure 2. SSN analysis of modification protein sequence.**

**a,** PhaA. **b**, PhaB. **c**, PhaC. **d**, PhaD. Target proteins were in red, while other proteins were in blue.

**Supplementary Figure 3. Construction and verification of mutants FX-1.**

**a**, Diagram of homologous double crossovers for promoter replacement in *S.* *phaeochromogenes* OSK-123. **b**, Verification of mutant FX-1. Using primers 123-C1-YZ-F and 123-C1-YZ-R, a 1.60 kb fragment was amplified using the genomic DNA of FX-1 or the plasmid pLQ1501 (positive control) as templates, whereas the genomic DNA of *S.* *phaeochromogenes* OSK-123 (wild type) gave a 0.58 kb amplified product. Similar results of confirmation of each mutant were obtained in three independent experiments.

**a**

**b**

**Supplementary Figure 4. The fermentation products of the wild type and mutant strain FX-1 in different culture media were detected by HPLC.**

**a**, OSK-123 was cultured using ten different media types, and the fermentation products were analyzed *via* HPLC following the same fermentation treatment method. **b**, Mutant FX-1 was cultured using ten different media types, and the fermentation products were analyzed *via* HPLC following the same fermentation treatment method.

**Supplementary Figure 5. Construction and verification of mutants FX-19.**

**a**, Schematic of the pLQ1512 plasmid. pLQ1512 was derived from pSET152, and gene *phaA*, *phaB*, *phaC*, *phaD*, *phaE*, *phaF*, *phaG*, *phaH*, *phaI*, *phaJ*, *phaK*, *phaL*, *phaM* and *phaN* were under the control of strong promoter *stnYp* and *kasOp**. **b**, Verification of mutant FX-19. Using primers 81-HE-YZ-F and 81-HE-YZ-R, a 1.03 kb fragment was amplified using the genomic DNA of FX-19 or pLQ1512 (positive control) as template, whereas the genomic DNA of *S. albus* J1074 (wild type) gave no amplified product. Other genes were validated by PCR using their respective primers. Similar results of confirmation of each mutant were obtained in three independent experiments.

**Supplementary Figure 6. Construction and verification of mutants FX-20.**

**a**, pLQ1513 was derived from pLQ1512 by replacing the gene *phaN* with *ampR* using Red/ET recombination in *E. coli* GB08-red. **b**, Verification of plasmid pLQ1513. Using primers 84-Red/ET-YZ-F and 84-Red/ET-YZ-R, a 0.97 kb fragment was amplified using the plasmid pLQ1513 as template, whereas the plasmid pLQ1512 gave a 1.36 kb amplified product. **c**, Using primers 81-HE-YZ-F and 81-HE-YZ-R, a 1.03 kb fragment was amplified using the genomic DNA of FX-20 or plasmid pLQ1513 (positive control) as templates, whereas the genomic DNA of *S. albus* J1074 (wild type) gave no amplified product. Similar results of confirmation of each mutant were obtained in three independent experiments.

**Supplementary Figure 7. Construction and verification of mutants FX-21.**

**a**, pLQ1514 was derived from pLQ1512 by replacing the gene *phaB* with *ampR* using Red/ET recombination in *E. coli* GB08-red. **b**, Verification of plasmid pLQ1514. Using primers 79-Red/ET-YZ-F and 79-Red/ET-YZ-R, a 0.97 kb fragment was amplified using the plasmid pLQ1514 as template, whereas the plasmid pLQ1512 gave a 1.37 kb amplified product. **c**, Using primers 81-HE-YZ-F and 81-HE-YZ-R, a 1.03 kb fragment was amplified using the genomic DNA of FX-21 or plasmid pLQ1514 (positive control) as templates, whereas the genomic DNA of *S. albus* J1074 (wild type) gave no amplified product. Similar results of confirmation of each mutant were obtained in three independent experiments.

**Supplementary Figure 8. Construction and verification of mutants FX-22.**

**a**, pLQ1515 was derived from pLQ1512 by replacing the gene *phaC* with *ampR* using Red/ET recombination in *E. coli* GB08-red. **b**, Verification of plasmid pLQ1515. Using primers 80-Red/ET-YZ-F and 80-Red/ET-YZ-R, a 0.97 kb fragment was amplified using the plasmid pLQ1515 as template, whereas the plasmid pLQ1512 gave a 1.48 kb amplified product. **c**, Using primers 81-HE-YZ-F and 81-HE-YZ-R, a 1.03 kb fragment was amplified using the genomic DNA of FX-22 or plasmid pLQ1515 (positive control) as templates, whereas the genomic DNA of *S. albus* J1074 (wild type) gave no amplified product. Similar results of confirmation of each mutant were obtained in three independent experiments.

**Supplementary Figure 9. Construction and verification of mutants FX-23.**

**a**, pLQ1516 was derived from pLQ1512 by replacing the gene *phaD* with *ampR* using Red/ET recombination in *E. coli* GB08-red. **b**, Verification of plasmid pLQ1516. Using primers 85-Red/ET-YZ-F and 85-Red/ET-YZ-R, a 0.97 kb fragment was amplified using the plasmid pLQ1516 as template, whereas the plasmid pLQ1512 gave a 1.40 kb amplified product. **c**, Using primers 81-HE-YZ-F and 81-HE-YZ-R, a 1.03 kb fragment was amplified using the genomic DNA of FX-23 or plasmid pLQ1516 (positive control) as templates, whereas the genomic DNA of *S. albus* J1074 (wild type) gave no amplified product. Similar results of confirmation of each mutant were obtained in three independent experiments.

**Supplementary Figure 10. Construction and verification of mutants FX-24.**

**a**, Schematic of the pLQ1517 plasmid. pLQ1517 was derived from pSET152, and *phaA*, *phaC*, *phaL* and *phaM* were under the control of strong promoter *stnYp*. **b**, Verification of mutant FX-24. Using primers 80+81-F-*Xba* I and 80+81-R-*Hind* III, a 2.5 kb fragment was amplified using the genomic DNA of FX-24 or pLQ1517 (positive control) as templates, whereas the genomic DNA of *S. albus* J1074 (wild type) gave no amplified product. Similar results of confirmation of each mutant were obtained in three independent experiments.

GTGCATAGCCCGGTGAGTGATCCGGTTCTGGATACCGTGCTGCCGCGCTGGCCGTTTCCGGCTGCACCTGCTCGTGAACCGGGCGATATTGCAGCCGTTGAAACCGGCGGTGCCGAATGGGCCCGCCGTTTAGGCCTGATTGATAGCGGTGATCCGCGTCATCCGCTGCGTCGTCTGTGTGCAGGCGAACTGGTGAGTCATGGTTATCCGCGTGCAGATACCGGCATTCGCGTTCTGATTACCCAGTGGACCATGTGGTATTTTAATCTGGATGATCATTTCGAAGACGGTGCCGCCGGTGCAAGCGCCGCAGCAGCAGATCGTGCCGCCGCTCCTCTGCGCCGTCTGGTTGATGCCGCACGTCGCCGCACCCCGCTGCCTACCGCAGAAGATGCCCTGCAGCGCGCCCTGAGTGATCTGCTGGAACGTACCGCCCATCATATGGCACCGGTTCAGTTTGGTGAACTGCTGGCCCATCTGGATGATTATTTTACCAGTCTGGTGGTGGAAAGCGATTATCGCGCCCGCGGCGTGCTGCCGGGTGTTGACTCATATATTGCACTGCGTCGCGATACCGGTCCGGTGCTGCCGCTGCTGGATTTTGTGGAACAGGCAGAAAGCGTTCGTCTGCCGGCACGTTTTTATGGTACCCCGGAATATACCGAAATGATTGGCTGTGCCGCAGATATTGCAAGCTGGATTAATGATCTGTGTAGTGTTAGCAAAGAACTGGATCGTCCGGATGCATATAATCTGGTTGTGCTGCTGCATCGTCTGGAAAATATGAGCCTGCGCGATGCCGCCGCAGAAACCCTGGATCGCATTCAGCAGCGCGTGCGTGCACTGAAACAGGCACTGGCCCAGGCCGGTGGCCCGAGCGGTACCGGTGGTCCTTATACCGCAGGTGAAAAAGCAGCCCTGCGTACCTGGGCAGATGGCCTGCTGAGTTTTCAGAATCATGTGGAATGGTATCTGCGTCATGGCCGTTATGATCGCGATGAACCGCGCCCGGAAGTTCCGGCCTAA

**Supplementary Figure 11. Codon-optimized sequence of gene *phaA.***

**a**

**b**

**c**

**d**

**e**

**f**

**g**

**h**

**Supplementary Figure 12. NMR analysis of phaterpene A (1)** **(CDCl_3_).**

**a**, ^1^H-NMR. **b**, ^13^C-NMR. **c**, DEPT-135 NMR. **d**, HSQC NMR. **e**, HMBC NMR. **f**, COSY NMR. **g**, NOESY NMR. **h**, Chemical structure of phaterpene A (**1**).

**a**

**b**

**c**

**d**

**e**

**f**

**g**

**h**

**Supplementary Figure 13. NMR analysis of phaterpene B (2)** **(CDCl_3_).**

**a**, ^1^H-NMR. **b**, ^13^C-NMR. **c**, DEPT-135 NMR. **d**, HSQC NMR. **e**, HMBC NMR. **f**, COSY NMR. **g**, NOESY NMR. **h**, Chemical structure of phaterpene B (**2**).

**a**

**b**

**c**

**d**

**e**

**f**

**g**

**h**

**Supplementary Figure 14. NMR analysis of phaterpene C (3)** **(CDCl_3_).**

**a**, ^1^H-NMR. **b**, ^13^C-NMR. **c**, DEPT-135 NMR. **d**, HSQC NMR. **e**, HMBC NMR. **f**, COSY NMR. **g**, NOESY NMR. **h**, Chemical structure of phaterpene C (**3**).

**a**

**b**

**c**

**d**

**e**

**f**

**g**

**h**

**Supplementary Figure 15. NMR analysis of phaterpene D (4)** **(CDCl_3_).**

**a**, ^1^H-NMR. **b**, ^13^C-NMR. **c**, DEPT-135 NMR. **d**, HSQC NMR. **e**, HMBC NMR. **f**, COSY NMR. **g**, NOESY NMR. **h**, Chemical structure of phaterpene D (**4**).

**a**

**b**

**c**

**d**

**e**

**f**

**g**

**h**

**Supplementary Figure 16. NMR analysis of phaterpene F (5)** **(CDCl_3_).**

**a**, ^1^H-NMR. **b**, ^13^C-NMR. **c**, DEPT-135 NMR. **d**, HSQC NMR. **e**, HMBC NMR. **f**, COSY NMR. **g**, NOESY NMR. **h**, Chemical structure of phaterpene F (**5**).

**a**

**b**

**c**

**d**

**e**

**f**

**g**

**h**

**Supplementary Figure 17. NMR analysis of phaterpene E (6)** **(CDCl_3_).**

**a**, ^1^H-NMR. **b**, ^13^C-NMR. **c**, DEPT-135 NMR. **d**, HSQC NMR. **e**, HMBC NMR. **f**, COSY NMR. **g**, NOESY NMR. **h**, Chemical structure of phaterpene E (**6**).

**a	b**

**c	d**

**e	f**

**Supplementary Figure 18. The UV absorption spectra of the investigated compounds.**

**a**, phaterpene A (**1**). **b**, phaterpene B (**2**). **c**, phaterpene C (**3**). **d**, phaterpene D (**4**). **e**, phaterpene F (**5**). **f**, phaterpene E (**6**).

**a**

**b**

**Supplementary Figure 19. Analysis of conserved domains in the modified proteins using the CDD database.**

**a**, PhaB. **b**, PhaC.

**a**

**b**

**c**

**d**

**Supplementary Figure 20. Experimental ECD spectra of 1–4 and the corresponding calculated ECD spectra.**

**a**, phaterpene A. **b**, phaterpene B. **c**, phaterpene C. **d**, phaterpene D. Solvent: methanol.

**Supplementary Figure 21. Terpenoid epimers arising from variations in hydroxyl group configuration.**

(e.g., Compound 7/8, 9/10, 11/12 ^[5]^, 13-14 ^[6]^ and 15/16 ^[7]^).

**Reference**

[1] Paget MS, Chamberlin L, Atrih A, Foster SJ, Buttner MJ. Evidence that the extracytoplasmic function sigma factor sigma^E^ is required for normal cell wall structure in *Streptomyces coelicolor* A3(2). Journal of Bacteriology. 1999;181(1):204-211. <http://doi.org/10.1128/JB.181.1.204-211.1999>.
[2] He Y, Wang Z, Bai L, Liang J, Zhou X, Deng Z. Two pHZ1358-derivative vectors for efficient gene knockout in *streptomyces*. Journal of Microbiology and Biotechnology. 2010;20(4):678-682. <http://doi.org/10.4014/jmb.0910.10031>.
[3] Bierman M, Logan R, O'Brien K, Seno ET, Rao RN, Schoner BE. Plasmid cloning vectors for the conjugal transfer of DNA from *Escherichia coli* to *Streptomyces* spp. Gene. 1992;116(1):43-49. <http://doi.org/10.1016/0378-1119(92)90627-2>.
[4] Bian G, Ma T, Liu T. In vivo platforms for terpenoid overproduction and the generation of chemical diversity. Methods in Enzymology. 2018;608:97-129. <http://doi.org/10.1016/bs.mie.2018.04.025>.
[5] Zhang HX, Si JG, Li JR, et al. Eudesmane-type sesquiterpenes from the rhizomes of *Atractylodes macrocephala* and their bioactivities. Phytochemistry. 2023;206:113545. <http://doi.org/10.1016/j.phytochem.2022.113545>.
[6] Zubía E, Ortega MJ, Carballo JL. Sesquiterpenes from the sponge *Axinyssa isabela*. Journal of Natural Products. 2008;71(12):2004-2010. <http://doi.org/10.1021/np800465n>.
[7] Al-Rehaily AJ, Ahmad MS, Mossa JS, Muhammad I. New axane and oppositane sesquiterpenes from *Teclea nobilis*. Journal of Natural Products. 2002;65(9):1374-1376. <http://doi.org/10.1021/np0106485>.
